# A study on metabolic characteristics and metabolic markers of gastrointestinal tumors

**DOI:** 10.1080/15384047.2023.2255369

**Published:** 2023-09-13

**Authors:** Shan Cong, shanshan Bai, Minghao Zhang, yanfang Bi, yu Wang, shi Jin, hui He

**Affiliations:** a Department of Laparoscopic Surgery, the First Affiliated Hospital of Dalian Medical University, Dalian, Liaoning Province, China; b Department of Ultrasound, The First Affiliated Hospital of Dalian Medical University, Dalian, Liaoning Province, China; c Department of Vascular Interventional, Affiliated Hongqi Hospital of Mudanjiang Medical College, Mudanjiang, China; d Department of Nursing, The First Affiliated Hospital of Dalian Medical University, Dalian, Liaoning Province, China

**Keywords:** Gastrointestinal tumor, metabolic subtype, prognosis, drug response, TCGA database

## Abstract

Tumor cells have significant heterogeneity in metabolism and are closely related to prognosis, gene mutation, and subtype. However, this association has not been demonstrated in reports of gastrointestinal tumors. In this study, we constructed four metabolic subtypes and identified four gene signatures using the expression data and clinical information of 252 metabolism-related genes from TCGA and NCBI databases for gastric adenocarcinoma (STAD) and colorectal cancer (COAD and READ). MC1 had the worst prognosis compared to other classifications. GSig1 was mainly related to drug metabolism and was the highest in MC1 with the worst prognosis, while the other subtypes were mainly related to glucose metabolism pathways. This difference also existed in other different malignant tumors. In addition, metabolic typing was associated with chemotherapeutic drug response and tumor heterogeneity, which indicated that monitoring metabolic typing could contribute to drug efficacy and gene-targeted therapy. In conclusion, we identified differences among subtypes in clinical characteristics such as prognosis and revealed the potential function of metabolic subtype in response to chemotherapeutic agents and oncogene mutations. This work highlighted the potential clinical meaning of metabolic subtype and characteristics in drug therapy and prognosis assessment of malignant tumors.

## Introduction

Malignant tumor has become one of the most common serious diseases that threaten human life and affect human quality. According to statistics, 18.1 million new cases and 9.6 million deaths from cancer globally in 2018, and approximately half of those were in Asia.^1^ Among them, gastric cancer has become the fifth most common cancer and the third leading cause of cancer death, while colorectal cancer has accounted for one-tenth of the total number of cancer deaths. It is expected that 21.4 million new cases and 13.2 million deaths will be related to malignant tumors by 2030.^2^ Similarly, the number of new cases and deaths of cancer in China shows an upward trend in recent years, and the age of onset is significantly younger.

In addition to radical surgical resection, chemotherapy remains one of the practical and adjunctive treatments for gastrointestinal tumors, especially for patients with difficult resection and extensive metastases.^3,4^ Although significant progress has been made in tumor biology research and treatment in recent years, there are still great limitations in the current clinical chemotherapy drugs. For example, various first-line antitumor drugs have been reported to have acquired drug resistance in tumor patients after long-term use, including bevacizumab and oxaliplatin.^5,6^ Moreover, most traditional chemotherapeutic drugs inhibit tumor cells and kill adjacent normal cells, with significant toxicity and severe side effects. The specific targeting drugs developed in recent years were less toxic, but the scope of application was relatively narrow, and tumors were prone to relapse after treatment. The main reason for the failure of targeted therapies for tumors is that most cancer cells have multiple genetic mutations or abnormalities. Targeted therapies for a specific gene molecule are not effective in killing tumor cells in most cases. Therefore, exploring the biological characteristics of tumor cells which are familiar but different from normal cells, and carrying out specific interventions according to these characteristics are the keys to improving the curative effect of tumor treatment.

Previous studies in pan-cancer tumor analyses based on global metabolic pathways have shown significant metabolic heterogeneity among tumors, closely related to prognosis, somatic driven mutation, and tumor subtype.^7–9^ The gastrointestinal tract is a vital structure associated with a metabolism, so that this association may be even more vital in gastrointestinal tumors. However, the heterogeneity of metabolic pathways and the relationship of metabolic pathways with the molecular subtypes and prognosis have not been reported in detail in gastrointestinal tumors.

This study collected and explored 252 gastrointestinal tumor metabolism-related genes and corresponding clinical information from TCGA and NCBI datasets. The consistency matrix was constructed to cluster the gastrointestinal cancer samples and establish four metabolic subtypes. We found that the distribution of clinical characteristics such as age, stage, and histological type among MClusters showed significant bias. At the same time, it was observed that the gene expression in different MClusters was significantly different and correlated with the prognosis. Next, we compared the distribution of patient response after treatment with commonly used antitumor drugs on different MC clusters. Finally, we assessed the metabolic characteristics and prognostic predictive ability of four metabolism-related gene signatures in numerous cancers and demonstrated the differences in metabolic subtypes’ genomic mutation characteristics and heterogeneity. These metabolic subtypes may show specific characteristics in prognostic and driver mutations.

## Methods

### Data source and pre-processing

GDC API in the TCGA database downloaded from University of California Santa Cruz (UCSC) was used to get the latest clinical follow-up information and corresponding STAD, COAD, and READ gene expression data (October 15, 2021). We downloaded GSE17536, GSE15459, GSE39582, and GSE62254 data from National Center for Biotechnology Information (NCBI), containing 177, 192, 585, and 300 samples, respectively. The following steps were performed for pretreatment: 1) Remove samples with no clinical data or overall survival (OS) of less than 30 days. 2) Remove samples of normal tissue. 3) Remove the gene with a transcript per million (TPM) <0.01 in half the samples.

Pre-process the data of GSE17536, GSE15459, GSE39582, and GSE62254 by the following steps: 1) The RMA method was used to homogenize all raw data. 2) The batch effect was removed using the batch correction method of LIMMA (Fig. S1). Then the pre-processed data was processed in the following steps: 1. Normal tissue samples were removed, and only tumor tissue data was retained. 2.Remove samples with no clinical information or an OS<30 days. 3) Use a Bioconductor package to map chip probe to human gene symbol (Table S1). Metabolism-related genes were derived from KEGG and Reactome databases. We downloaded genes corresponding to pathways containing keywords such as carbohydrates, oxidations, glycogen, glycogenolysis, glycolysis, pyruvate, citric, fatty acid, and mitochondrial from KEGG and Reactome databases, respectively. Then, the duplicated genes between the pathways were removed, and a total of 252 genes that co-existed between the two databases were obtained (Table S2).

### Identification of metabolic subtypes and metabolic gene signatures

Firstly, we extracted the expression levels of 252 metabolism-related genes, and 209 genes were obtained after removing the low expression levels. The consensus cluster method was used to construct the consensus matrix based on the expression levels of 209 genes, and the cluster typing of gastrointestinal cancer samples was performed. In order to obtain the optimal clustering results, we evaluated the clustering results with K values ranging from 2 to 10. According to consensus index, consensus cumulative distribution function (CDF) curve, and Delta area distribution (relative change in area under CDF curve), the optimal number of clusters was four on both TCGA and GEO cohorts (Fig. S2), defined as MC1, MC2, MC3, and MC4 respectively. Next, the R Package random Forest algorithm was used to evaluate the importance of 252 metabolism-related genes. We set the number of random variables (mtry parameter) of each partition as 1 to 74 with ntree = 500 and selected the mtry value with the lowest error rate as the optimal mtry value of the Random Forest algorithm.

According to the error rate of random Forest, ntree = 200 was selected. Finally, the metabolic genes were sorted according to their importance, and a total of 169 genes with cumulative importance > 95% were selected for subsequent analysis (Fig. S3). According to gene expression levels of 169 genes on four MClusters, we identified a group of genes as the signature of a MCluster if the genes had the highest expression levels in the MCluster. Therefore, 169 genes were divided into four groups as the gene signatures of each MCluster, where a total of 65 gSig1 genes, 36 gSig2 genes, 34 gSig3 genes, and 34 gSig4 genes were identified in MC1, MC2, MC3, and MC4, respectively.

### Drug response analysis of metabolism-related genes

Information on drug therapy came from clinical data from the TCGA samples. The TCGA recorded the patients’ information and related therapeutic drugs and provided the patients’ disease progress after drug treatment, including progressive clinical disease, stable disease, complete response, and partial response. In addition, the response data of tumor cell lines were derived from Genomics of Drug Sensitivity in Cancer (GDSC). The database records IC_50_ data for nearly 1,000 human tumor cell lines responding to more than 300 drug compounds.

### SSNV analysis of the genome

The SSNV data was obtained from the TCGA database (https://www.cancer.gov/about-nci/organization/ccg/research/structural- genomics/tcga, version mutect2), and the mutation data are whole-exome sequencing. In tumor mutation load (TMB) analysis, the silent and intron interval mutations were first removed, and the genomic interval was 38.4Mb. Then the TMB of each sample was the number of all mutations divided by 38.4Mb.

### Statistical analysis

**Figure 1.**
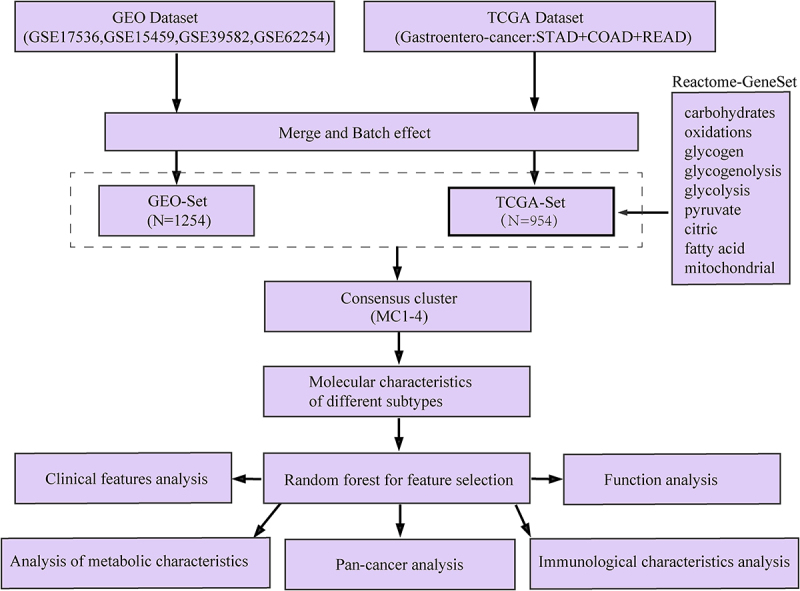
The flow chart of the research process.

Cox regression models were used to assess the relationship between gene module scores and prognosis (OS and PFS), and *p* < .05 was the significance threshold in the log-rank test. In addition, the chis-q test was used to test the significance of sample overlap between histological types and MClusters and the distribution bias of clinical characteristic group samples on MClusters. Wilcox rank test was used to test the significance between two groups of continuous variables, while Kruskal Wallis rank test was used for the significance test of more than two groups. The BH method calculated the false discovery rate (FDR). All of the above analysis was done using R (version 3.5.1). Unless otherwise specified, * * * represented *p* < 1×e^−3^, * * represented *p* < .01 and * represented *p* < .05. The whole research process was showed in Figure 1.

## Results

### Metabolic typing analysis of gastrointestinal carcinoma

**Figure 2.**
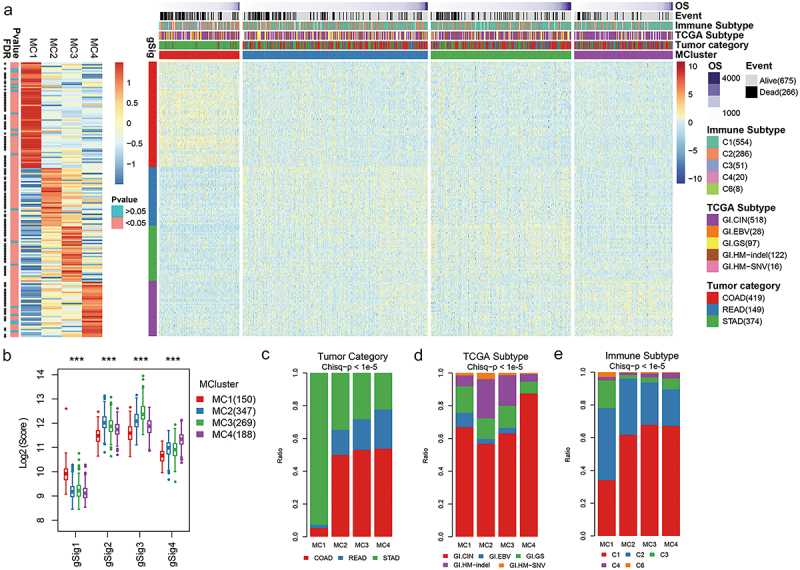
Molecular typing of gastrointestinal cancer based on metabolism-related genes.

The consistent clustering algorithm was used to cluster gastrointestinal cancer samples in the TCGA database downloaded from UCSC, and the optimal number of clusters was 4, which were defined as MC1 (150), MC2 (347), MC3 (269), and MC4 (188), respectively (Figure 2A). According to the expression pattern of genes in MClusters, four gene modules were obtained, which were gSig1 (65), gSig2,^10^ gSig3,^11^ and gSig4.^11^ Among them, gSig1 had the highest expression level in MC1, and the expression level in other subtypes was generally low. The expression of gSig2, gSig3, and gSig4 was higher in MC2, MC3, and MC4, while their expression was lowest in MC1 (Figure 2B). The significance test showed that 151 of the 169 genes differed significantly in MCluster expression (Table S3, p &lt;0.05). By comparing the composition of tumor types in MClusters, we found that MC1 was mainly composed of STAD samples and included a small number of COAD samples, while READ samples were the least. However, the distribution of these three types of samples was more uniform in MC2, MC3, and MC4 (Figure 2C). The distribution of pathological subtypes from the TCGA database in MClusters also showed a significant bias (Figure 2D, chis-q *p* < 1×e-5). For example, GI.HM-indel samples were mainly distributed on MC2 and GI.CIN samples were mainly distributed on MC4. Among the immune typing samples based on immune characteristics, the samples of C2, C3, and C6 immune subtypes were mainly concentrated in MC1, while the distribution of other MClusters was less (Figure 2E).

**A**: Heatmap of expression levels of metabolic subtypes and gene signatures in gastrointestinal cancer. The left panel was the heatmap of the average expression level of 169 genes on MClusters. **B**: The distribution of gene signature fraction on MClusters. **C**: Comparison of tumor composition of metabolic subtypes. **D**: Comparison of metabolic subtypes and pathological subtypes. **E**: Comparison of metabolic subtypes and immune subtypes.

### Analysis of metabolic gene module characteristics

**Figure 3.**
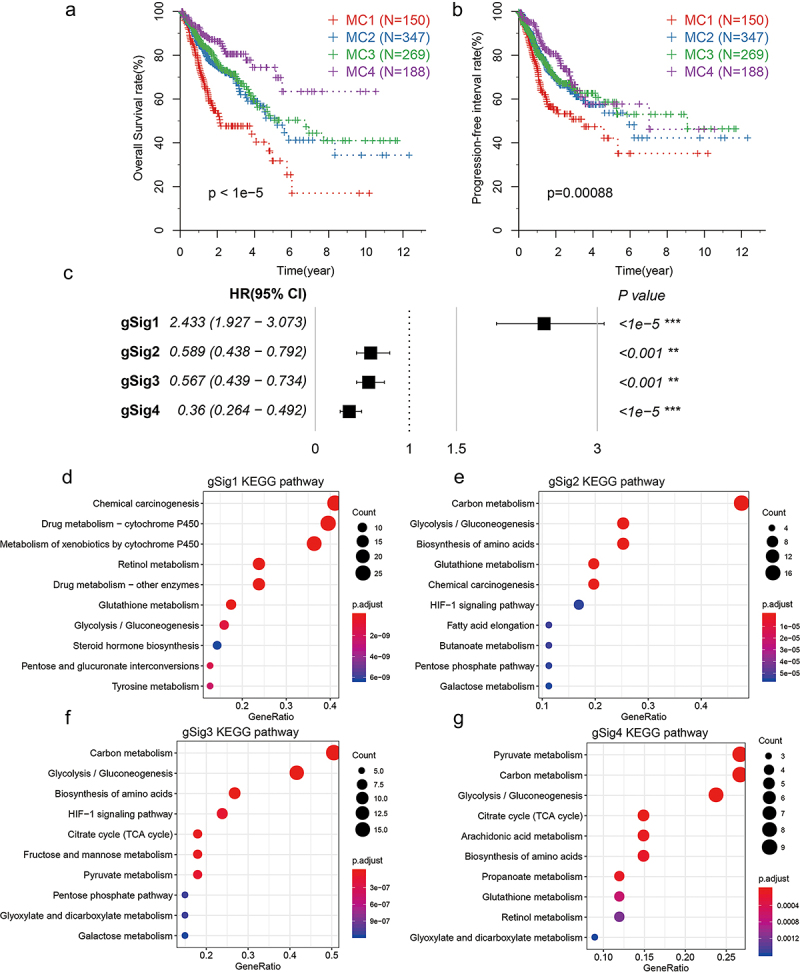
Expression characteristics and functional annotation of gene signatures.

By comparing the distribution of samples with different clinical characteristics in MClusters, we observed that samples with age, stage, and histological type among MClusters differed significantly (Table S4). The OS analysis showed that there were significant differences between MClusters (Figure 3A). MC1 had the worst prognosis, MC4 had the best prognosis, while MC2 and MC3 had a prognosis between them. The progression-free survival (PFS) among MClusters also showed a significant difference, with a trend consistent with OS (Figure 3B). The analysis of gene signature scores found that they were significantly related to OS. GSig1 was an adverse prognostic factor, while gSig2, gSig3, and gSig4 were favorable prognostic factors (Figure 3C). GO enrichment analysis of gene modules showed that gSig1 was mainly related to drug metabolism (Figure 3D), while gSig2, gSig3, and gSig4 were all related to glycolysis and gSig2 metabolic pathways (Figure 3E-G), which reflected a significant difference between gSig1 and the other three. In conclusion, although gSig2, gSig3, and gSig4 have different expression levels in different MClusters, they are highly similar in function.

**A-B**: KM curves of OS(A) and PFS(B) for metabolic subtypes in the TGGA dataset. **C**: Relationship between gene signature score and OS prognosis. **D-G**: The KEGG pathway enrichment results of gSig1(D), gSig2(E), gSig3(F), and gSig4(G). The dot plot showed the top 10 pathways.

### Metabolic typing of GEO independent validation set

**Figure 4.**
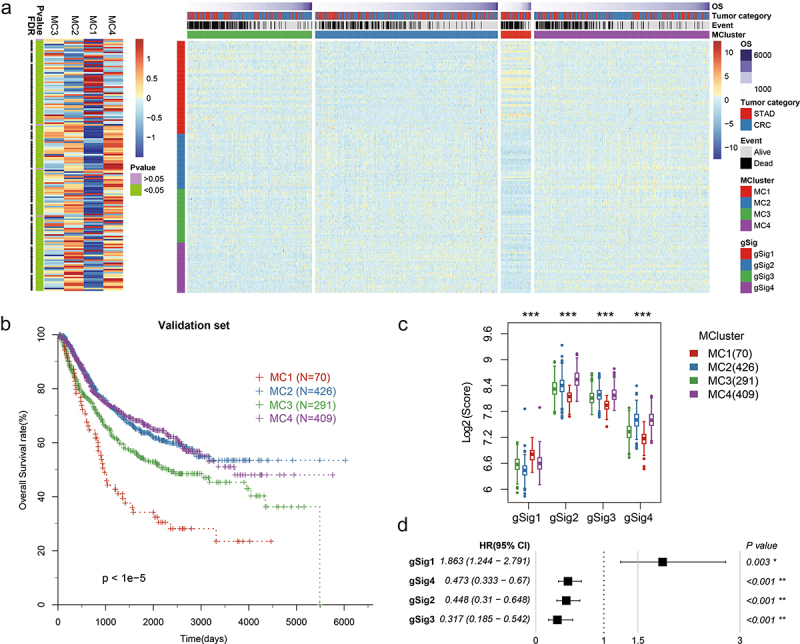
Classification of metabolic subtypes in independent validation sets.

In the independent GEO dataset, we used 169 metabolism-related genes to perform cluster analysis on 1196 gastrointestinal cancer samples and obtained the optimal cluster number of 4 (Fig. S1.D-F). The expression of four gene modules in MClusters showed significantly different characteristics (Figure 4A). Notably, in the MC1 subtype with the worst prognosis, gSig1 expression was significantly higher than that in the other subtypes (Figure 4B-C), which was consistent with the results of metabolic typing based on the TCGA dataset. The relationship between gene signature score and prognosis showed the same trend as the TCGA dataset (Figure 4D).

**A**: Heatmap for verifying the expression level of metabolic subtypes and gene modules in gastrointestinal cancer. **B**: The OS distribution for verifying metabolic subtypes of gastrointestinal cancer. **C**: Distribution of gene signature scores in different metabolic subtypes. **D**: Relationship between gene module score and OS.

### Analysis of the differences in metabolic pathways among the four subtypes

**Figure 5.**
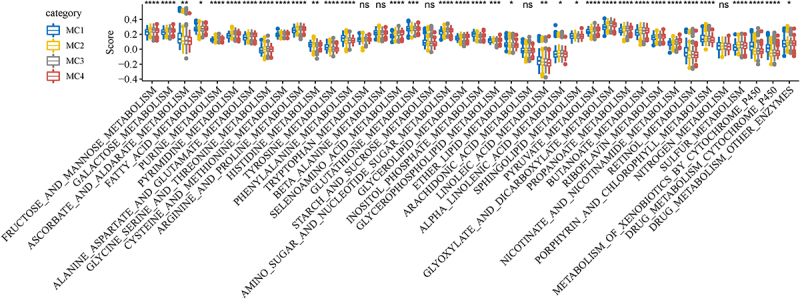
The different distribution of metabolic pathways among the four subtypes.

To further explore the differences in metabolic pathways among the four subtypes, we evaluated the enrichment scores of 40 metabolic pathways in the KEGG database using the ssGSEA and calculated the distribution differences of these 40 metabolic pathways in MC1, MC2, MC3 and MC4. And we can observe that a lot of the metabolic pathways have significant differences (Figure 5).

### Analysis of metabolic subtypes and drug response

**Figure 6.**
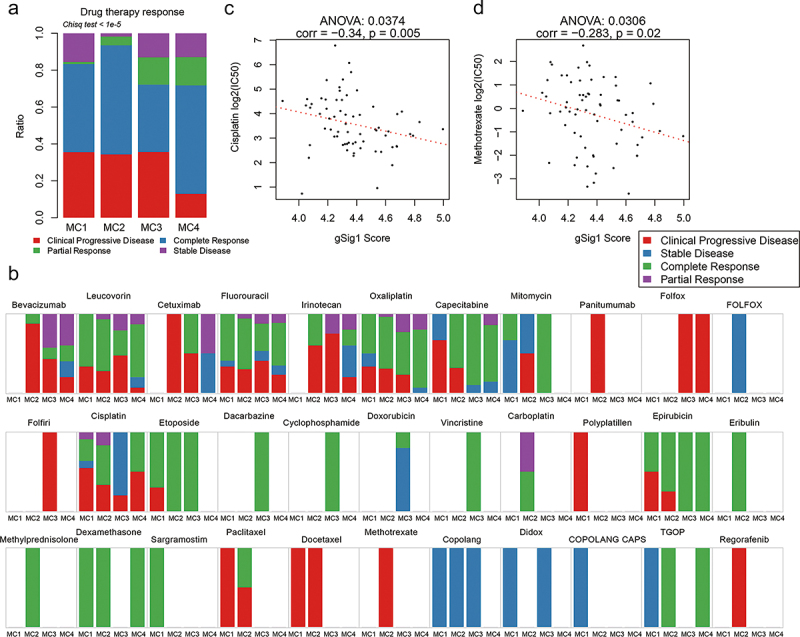
Drug response analysis of metabolic subtypes.

The results of gene signature annotation showed that gSig1 was involved in drug metabolism. Considering that the gSig1 score was an unfavorable factor in both TCGA and GEO datasets, we speculate that the imbalance of some gsig1 genes involved in drug metabolism may interfere with the metabolism of related drugs, reduce the response and sensitivity of patients to drugs, and lead to poor prognosis. First, we compared the distribution of prognosis from patients treated with antitumor drugs on MClusters. Although the MC1 subtype with the worst prognosis consisted mainly of patients with a complete response and stable disease types, we observed that the distribution of patients with progressive clinical disease in MC4 with the best prognosis was significantly lower than that in MC1, MC2, and MC3 (Figure 6A). Meanwhile, the drug response time of MC1 patients was significantly lower than the other three groups (Fig. S4).

**A**: Distribution of drug treatment response types in MClusters. **B**: Distribution of responses of 33 therapeutic drugs in MClusters. **C-D**: The relationship between IC_50_ and gSig1 scores of gastrointestinal cancer cell lines in response to Cisplatin(C) and Methotrexate(D).

Further analysis of drug response on MClusters showed that 19 out of 33 drugs showed disease progression after treatment, and these patients with progression were mainly in MC1, MC2, and MC3 with poor prognosis (Figure 6B, Fig. S5). Based on the data of tumor cell lines responding to drugs provided by the GDSC database, we analyzed the relationship between IC_50_ of Cisplatin and Methotrexate (no corresponding data for other drugs) and the gSig1 score of gastrointestinal cancer cell lines. Then we observed a significant negative correlation between the gSig1 score and IC_50_ of both drugs (Figure 6C-D), while no negative correlation was observed from gSig2, gSig3, and gSig4 (Fig. S6). These results suggest that patients with high gSig1 scores may be sensitive to cisplatin and methotrexate.

### Analysis of Pan-cancer characteristics in metabolism-related genes

**Figure 7.**
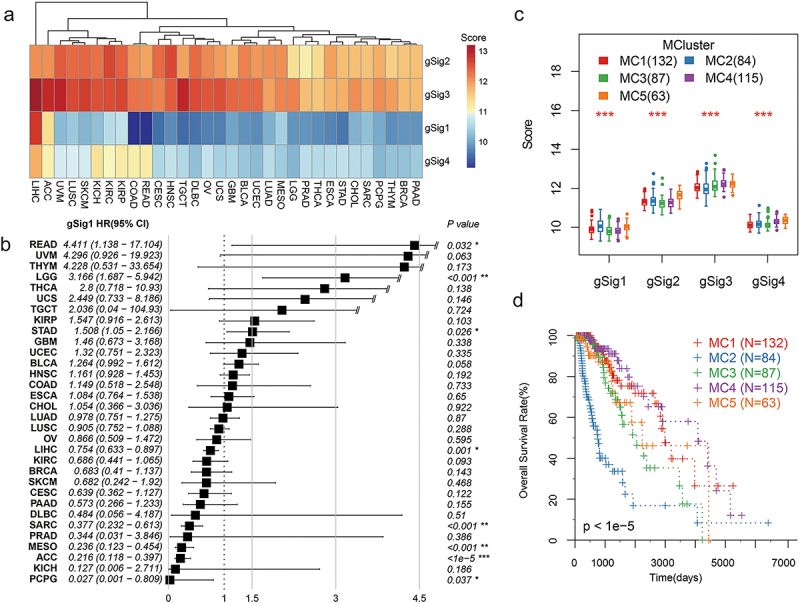
Characteristic analysis of metabolism-related genes in pan-cancer.

The TCGA database provided data on 32 broad categories of cancer molecules. We calculated the scores of four metabolism-related gene signatures for these cancers and observed that gSig1 and gSig4 scores were significantly lower than gSig2 and gSig3 scores for all cancers (Figure 7A). Interestingly, among the 32 cancers, COAD and READ had the lowest gSig1 scores, reflecting the low drug metabolic activity of colorectal cancer, which was significantly different from liver hepatocellular carcinoma (LIHC) and adrenocortical carcinoma (ACC) (Table S5). The prognostic analysis showed that the gSig1 score was a significant adverse prognostic factor in three cancers, including READ, STAD, and brain lower-grade glioma (LGG). Although the gSig1 score of COAD was not significantly associated with prognosis, its hazard ratio (HR) above one also indicated that gSig1 implied an unfavorable prognosis (Figure 7B). Interestingly, the gSig3 score of LGG also showed a significant favorable prognostic factor, suggesting that LGG may have similar metabolic characteristics to gastrointestinal cancer (Fig. S7). The metabolic typing of LGG further helped us confirm the above results. We observed that LGG could be divided into five optimal metabolic subtypes (Fig. S8). There were also significant prognostic differences among the subtypes, and the unfavorable prognostic factor gSig1 had the highest score in the MC2 subtype with the worst prognosis (Figure 7C-D).

**A**: The distribution of gSig1 score in 32 carcinomas. **B**: The HR of gSig1 on 32 carcinomas. **C**: The distribution of gene signature scores in LGG metabolic subtypes. **D**: KM curves of OS form LGG metabolic subtypes.

### Genomic heterogeneity analysis of metabolic subtypes

**Figure 8.**
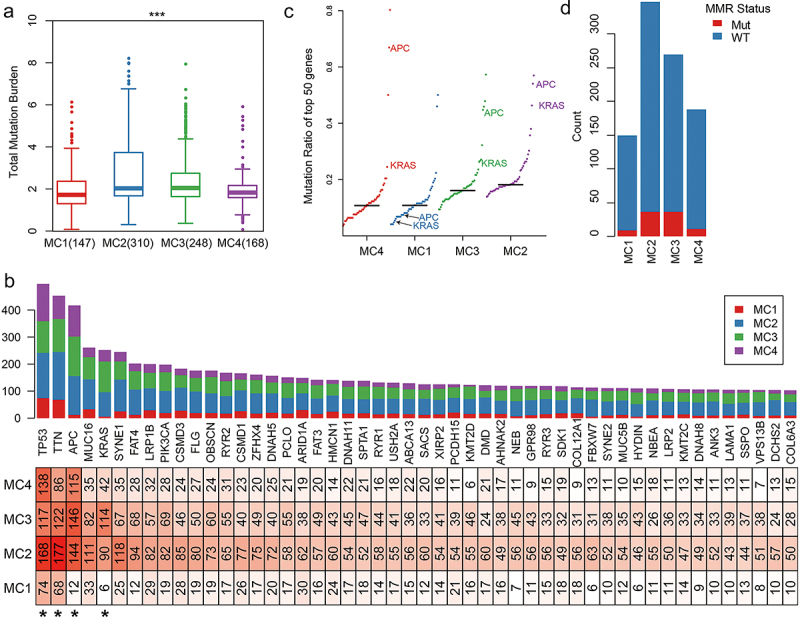
Genomic mutation characterization of metabolic subtypes.

For the metabolic subtypes of gastrointestinal cancer, we analyzed the genomic characteristics between MClusters. On the whole, MC1 and MC4 had low mutation load, while MC2 and MC3 had high mutation load (Figure 8A, Numbers of median mutations in the sample: 88/116/115.5/97). Tumor suppressor gene TP53 had the highest mutation rate in all MClusters. In addition, TTN, APC, KRAS, PIK3CA, and other genes also had high mutation rates in MClusters (Figure 8B). Although the mutations of these genes were high among the samples, the mutations of APC and KRAS in MC1 were significantly lower than those in MC2, MC3, and MC4, reflecting the preference of mutations in the different metabolic subtypes (Figure 8C). Considering the vital influence of the DNA mismatch repair (MMR) system on genomic mutations, we compared the mutations of five MMR-related essential genes, including MLH1, MSH2, MSH6, PMS2, and EPCAM, in MClusters. The mutation rate of MC2 and MC3 with high mutation load was significantly higher than that of MC1 and MC4 (Figure 8D, *p* = .013). The frequency of mutations in metabolism-related genes was also significantly higher in MC2 and MC3 subtypes than in MC1 and MC4. Overall, they mutated less frequently in the gastrointestinal cancer genome and in other cancers (**Fig. S9**).

**A**: The TMB distribution of metabolic subtypes. **B**: The numbers of top 50 gene mutations in metabolic subtypes. * Indicated that there were significant differences in gene mutations between MClusters (FDR <0.05). **C**: The proportion of mutations in the top 50 genes in different metabolic subtypes. **D**: MMR gene mutations in metabolic subtypes.

### The relationship between the metabolic subtypes and immunotherapy

**Figure 9.**
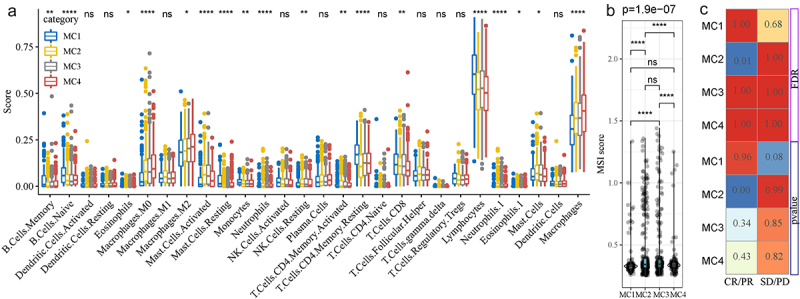
The relationship between the 4 metabolism subtypes and immune.

Immune cells in the tumor microenvironment played an important role in the tumor growth.^12^ We further explored the different distribution of 28 immune cells in the 4 metabolic subtypes,^13^ which showed that a lot of immune cells were significantly differently distributed in MC1, MC2, MC3 and MC4 (Figure 9A). What’s more, we compared the Microsatellite Instability (MSI) among the 4 metabolic subtypes using the MSI data from the previous study,^14^ as we can see (Figure 9B), the scores of MSI in MC1 were significantly lower than them in MC2 and MC3. Finally, we analyzed the response to immunotherapy in the 4 metabolic subtypes using the data immune checkpoint inhibitor (ICI) therapy datasets^15^ by mapping four metabolic subtypes into immunotherapy cohorts through the submap, which showed that MC2 was significantly associated with the response to immunotherapy (Figure 9C).

**A**: The different distribution of 28 immune infiltrating cell scores among the four subtypes. **B**: MSI scores among the four metabolic subtypes. **C**: The significance of potential benefit of immunotherapy with four molecular subtypes.

## Discussion

Gastrointestinal tumors are a common type of malignant tumor with high incidence rate and mortality rates, such as STAD, COAD, and READ. According to statistics, gastric cancer incidence and death cases ranked sixth and second, respectively, in the total number of malignant tumor patients globally in 2018. Colon cancer ranked fourth and fifth, and rectal cancer ranked eighth and tenth, respectively.^1^ Due to the lack of accurate biological characteristics or biomarkers, it is hard to provide timely evaluation and prediction of the treatment and prognosis targeting gastrointestinal tumors, and the five-year survival rate is only less than 5%.^16^ Previous reports have confirmed significant heterogeneity in metabolism among tumors, including lung cancer, LIHC, and brain tumors,^17–19^ but no relevant analysis and reports were conducted on gastrointestinal tumors. Based on this heterogeneity, we used STAD and colorectal cancer data from TCGA and GEO databases to analyze further the characteristic changes in metabolism-related pathways of gastrointestinal tumors. Firstly, we constructed four metabolic subtypes of gastrointestinal cancer by screening 252 metabolism-related genes. There were significant differences in OS and PFS among the subtypes, with the MC1 subtype having the worst of both. This result suggested that metabolic subtypes can be used as a reliable and independent prognostic biomarker to assess patient outcomes.

Next, we divided the 169 screened key genes into four signatures according to their expression levels, including gSig1, gSig2, gSig3, and gSig4. Functional annotation of gene modules showed that gSig1 was mainly related to drug metabolism and the expression was highest in the MC1 subtype with the worst prognosis, while gSig2, gSig3, and gSig4 were mainly related to glycolysis and gluconeogenesis metabolic pathways. Since the rapid proliferation of tumor cells requires energy, the overexpression of glycolysis-related genes has been confirmed to play a key role in the invasion and metastasis of malignant tumors.^20,21^ For example, Glucosamine-6-phosphate isomerase 1 (GNPDA1) catalyzes the formation of fructose 6-phosphate, a glycolytic raw material, thereby promoting the glycolysis pathway and cancer progression, including HCC, pancreatic cancer, and colorectal cancer.^22–24^ Similarly, we observed a high mutation frequency of the proto-oncogene KRAS on MC clusters, regulating the glucose metabolic pathway to support cancer cell proliferation.^25^ In addition, lactic acid, one of the critical products of the glycolysis pathway, also can promote VEGF and assist the immune escape of tumor cells. Based on these, there have been some exciting results in recent years in targeting tumors by inhibiting the activity of critical enzymes in glycolysis. The proliferation of tumor cells was briefly controlled and even apoptosis.^26^ In our study, glucose metabolism-related pathways, including glycolysis and gluconeogenesis, were enriched in gene signatures with a relatively good prognosis, consistent with previous studies. Meanwhile, samples of the independent validation set from the GEO database could also be divided into four metabolic subtypes with significant differences in prognosis. The worst prognostic subtype also had the highest expression levels of drug metabolism-related gSig1, suggesting that our constructed metabolic subtypes were reproducible.

Based on the above results, we found that the expression of gSig1 related to drug metabolism was highest in the subtype with the worst prognosis. Therefore, we speculated that the abnormal expression of drug metabolism-related genes might be associated with a poor prognosis. The drug response analysis of the TCGA dataset also confirmed our prediction. Overall, the MC1 subtype with the worst prognosis had the shortest drug response time. The progression samples were mainly concentrated in MC1, MC2, and MC3 with poor prognosis, while it was rarely distributed in the MC4 subtype with the best prognosis. For example, oxaliplatin is a classic first-line chemotherapy drug for malignant tumors such as COAD and READ, which can promote apoptosis of tumor cells by activating p53, upregulating the expression of Bax, and inducing the activation of caspase 3.^27,28^ However, it has been reported that cells gradually develop resistance to oxaliplatin during antitumor therapy, especially in patients with metastases.^29^ This phenomenon may be related to reduced drug accumulation and miRNA expression imbalance in drug-resistant cells.^30,31^ In our study, the gSig4 subtype with the best prognosis had the lowest drug progression to oxaliplatin. It may be because gSig4 contained the least patients who had developed to stage IV, which means less malignant tumor metastasis and less drug resistance. In addition, the same phenomenon has been observed in bevacizumab. Bevacizumab is a class of anti-VEGF monoclonal antibodies used in tumor immunotherapy that was prone to acquired drug resistance.^32^ When combined with capecitabine, the drug resistance of monotherapy was reversed.^33^ In the gSig4 subtype, bevacizumab and capecitabine had the fewest samples with disease progression after treatment, further suggesting that the metabolic subtype is a reliable and independent prognostic indicator.

The abnormal metabolism of tumor cells, especially the abnormal drug metabolism, has an important impact on treatment prognosis. In gastrointestinal tumors, we also found the lowest expression level of gSig1 in 32 malignant tumors and higher expression of other signatures, which was closely related to the rapid proliferation and expansion of tumor cells. The lower score of gSig1 and gSig4 in all involved malignancies also indicated the universality and unity of gene signature in malignant tumors. The lowest scores of COAD and READ in the analysis of the pan-cancer characteristics revealed the prevalence of low drug metabolic activity and high drug resistance in colorectal cancer, especially in patients with poor prognoses. Previous studies have shown that resistance to chemotherapeutic agents in rectal malignancies significantly reduces the effectiveness of cancer treatment and the quality of patient outcomes.^34^

Interestingly, the adverse prognostic role of gSig1 and the favorable prognostic role of gSig3 were also observed in LGG, suggesting that LGG and gastrointestinal tumors may have similar biological metabolisms. The typing of LGG further confirmed that the expression level of gSig1 was highest in the subtype with the worst prognosis. Few previous reports on this subject have tested our hypothesis. Sun *et al*. and Lin *et al*. have proposed that DNA abnormalities and epigenetic degeneration were present in various malignancies, including gastrointestinal tumors and LGG.^11,35^ In addition, the MIR155 gene was highly expressed in cholangiocarcinoma and LGG abnormally, which affected the prognosis of patients by mediating immune cell infiltration and immune checkpoint expression.^36^ This suggested that there may be common metabolic pathways and gene expression between gastrointestinal tumors and LGG to mediate the occurrence and development of tumors.

In addition, metabolic subtypes also showed significant differences in genomic mutation characteristics. MC1 and MC4 had the lowest mutation load, while MC2 and MC3 had the highest mutation load. Through analysis, tumor suppressor gene TP53 and oncogenes TTN, APC, KRAS, and PIK3CA showed the highest mutation frequency on MClusters. It is well known that abnormal methylation of tumor suppressor genes and abnormal demethylation of oncogenes are critical mechanisms in the induction and progression of malignant tumors. TP53 is a common missense mutation gene in malignant tumors with significant carcinogenic potential.^10,37^ TP53 gene is responsible for encoding tumor suppressor protein p53. Its mutation will lead to the inactivation of tumor mechanisms such as chromosome stability, DNA repair, and apoptosis.^37,38^ We observed that metabolic subtypes with poor prognosis, including MC1 and MC3, had the lowest TP53 mutations. However, Li *et al*. reported that the antitumor ability of TP53 mutant samples was significantly lower than that of TP53 wild type in STAD and COAD.^39^ This difference may be due to the breaking of the balance between the TMB and the tumor aneuploidy level (TAL) on tumor immunity caused by a gene mutation. In addition, KRAS mutations are common in more than 80% of pancreatic cancer cases and more than 30% of colorectal cancer cases.^40^

Interestingly, simultaneous mutations of KRAS and TP53 were observed in 40% of tumor samples, while simultaneous mutations of KRAS and PIK3CA were relatively rare, only in less than 10% of samples.^41^ Besides interfering with glucose metabolism pathways, Cheng *et al*. found that KRAS mutations were positively associated with high Treg cell infiltration in malignant tumor tissues, which secreted cytokines to inhibit cytotoxic CD8^+^ T cell function and promote the immune escape of tumor cells, ultimately leading to poor prognosis.^42,43^ Although mutation rates were low in gastrointestinal tumor samples, there were significant differences in the distribution of mutation frequencies of metabolism-related genes in different prognostic subtypes. Similar phenomena also appeared in the character analysis of other malignant tumors, which was related to the fact that they are critical genes involved in cells’ most fundamental biological processes. The inactivation of these metabolism-related genes will significantly affect cell survival and is also the basis for monitoring the occurrence and development of tumors.

By the way, the metabolism subtypes were also associated with the immunotherapy. ICI therapy has been a very effective therapy for patients with metastatic colorectal cancer (mCRC) that is mismatch-repair-deficient (dMMR) or microsatellite instability-high (MSI-H).^44^ However, there were not many specific biomarkers to predict responsiveness of immunotherapy. Among the metabolism subtypes, MC2 was high MSI and with a satisfactory response to ICI therapy, at the same time, MC1 was low MSI with a poor response to ICI therapy. Thus, our four metabolic subtypes may provide a new perspective to assess immunotherapy.

Nevertheless, our research was not complete and had some limitations. Studies of gastrointestinal tumors with three types of samples are rich in sample size but can lead to masking and overlapping differences between samples, including prognosis, drug metabolism, and immune infiltration. The combination therapy of chemotherapy drugs is now widely used in clinical antitumor work, which can effectively avoid the resistance of individual drugs.^45,46^ The lack of tumor cell response to drug combination therapy will be a problem to be solved in the future. Meanwhile, the study on the common mutation frequency of tumor suppressor genes and oncogenes is also worth further research.

## Conclusions

In summary, we constructed four metabolic subtypes of gastrointestinal cancer samples and classified 169 metabolism-related genes into four gene signatures. Our study was the first to identify the differences in clinical characteristics among subtypes, including age, stage, and prognosis. Our study compared the responses of tumor cells to common chemotherapeutic agents and revealed the mutation frequency and potential of oncogenes in different subtypes. Anyhow, the heterogeneity and molecular subtypes of metabolic pathways promise to be independent and effective biological indicators for monitoring prognosis and treatment and to provide additional benefits for patients suffering from gastrointestinal malignancies.

## Supplementary Material

Supplemental Material

## Data Availability

The dataset used and analyzed during the current study is available from the corresponding author on reasonable request.
