# Supplementary material for: A study on metabolic characteristics and metabolic markers of gastrointestinal tumors
Source: Cancer Biol Ther. 2023 Sep 13;24(1):2255369. doi: 10.1080/15384047.2023.2255369 (PMC10503448; doi:10.1080/15384047.2023.2255369)
Supplement: Supplemental Material [file KCBT_A_2255369_SM1060.zip › Supplementary material/Supplementary figure and table legends.docx]

**Supplementary figure legends**

**Supplementary Figure S1.** The expression distribution of Affymetrix data after RMA processing. **A-D:** Results of RMA processing for GSE17536(A), GSE15459(B), GSE39582(C) and GSE62254(D) datasets. **E:** Results of batch correction for four datasets.

**Supplementary Figure S2.** Consistent clustering results of TCGA and GEO datasets. **A/D:** Consistency matrix of TCGA(A) and GEO(D) datasets at K=4. **B/E:** Consensus index distribution curves of TCGA(B) and GEO(E) datasets. C/F: Delta area values of TCGA(C) and GEO(F) datasets at K=2-10. The consensus index reflected the stability of sample clustering. Simply, a certain number of samples (usually 2) were randomly selected from the clustering results each time. A ratio can eventually be calculated by counting the number of times these samples come from the same class. The larger the value was, the more stable the sample cluster was. Delta area reflected the amplitude of area change under the consensus index curve at different K values. The greater the range of change, the greater the influence of K change on clustering results.

**Supplementary Figure S3.** Random forest importance for IRGs with mean decrease gini index (blue) and mean decrease accuracy (red). The figure shows the top 10 critical genes' symbols. The dashed lines correspond to genes with cumulative importance >95%.

**Supplementary Figure S4.** Drug response time distribution of metabolic subtypes.

**Supplementary Figure S5.** Results of drug responses to different metabolic subtypes. **A-D:** Results of common drug responses in MC1(A), MC2(B), MC3(C), and MC4(D) subtypes. The red boxes highlighted two drugs Cisplatin and Methotrexate, that have IC_50_ records on the GDSC database.

**Supplementary Figure S6. A-C:** The IC50 relationship among gSig2(A), gSig3(B), gSig4(C) scores, and Cisplatin. **E-G:** The IC50 relationship among gSig2(E), gSig3(F), gSig4(G) scores, and Methotrexate. GSig scores were calculated using expression profiles of gastrointestinal tumor cell lines. The IC_50_ of a drug on cell lines was obtained from the GDSC database.

**Supplementary Figure S7. A-C:** Relationship among gSig2(A), gSig3(B), gSig4(C) scores and prognosis. The HR and P value were calculated by cox univariate regression analysis.

**Supplementary Figure S8. A:** Consistency matrix of LGG samples from the TCGA database at K=5. **B:** Consensus index curve when K=2-10. **C:** The expression levels of gene signatures in LGG samples at different metabolic subtypes.

**Supplementary Figure S9.** Mutation characteristics of metabolism-related genes. **A:** The distribution of mutations in 169 metabolism-related genes from different metabolic subtypes. **B:** The mutation ratio of 169 metabolism-related genes in different metabolic subtypes. **C:** The proportion of mutations in 169 metabolism-related genes from 32 carcinomas. The mutation ratio referred to the ratio of mutations in each subtype or cancer to all samples.

**Supplementary table legends**

**Supplementary Table S1:** Clinical characteristics of TCGA and GEO samples

**Supplementary Table S2:** The list of 252 metabolism-related genes from KEGG and Reactome databases

**Supplementary Table S3:** The differed expression of 151 genes in various MCluster subtypes

**Supplementary Table S4:** Comparison of the distribution of samples with various clinical characteristics in Mclusters

**Supplementary Table S5:** Analysis of the scores of four metabolism-related gene signatures in Pan-cancer
