# Supplementary material for: A study on metabolic characteristics and metabolic markers of gastrointestinal tumors
Source: Cancer Biol Ther. 2023 Sep 13;24(1):2255369. doi: 10.1080/15384047.2023.2255369 (PMC10503448; doi:10.1080/15384047.2023.2255369)
Supplement: Supplemental Material [file KCBT_A_2255369_SM1060.zip › Supplementary material/Table S1.docx]

**Table S1: Clinical characteristics of TCGA and GEO samples**

| **characteristics** | **TCGA-chort** | **GEO-chort** |
| --- | --- | --- |
| Event |  |  |
| Alive | 1352 | 1428 |
| Dead | 532 | 964 |
| Age |  |  |
| 0~50 | 206 | 298 |
| 50~60 | 364 | 428 |
| 60~70 | 544 | 730 |
| 70~100 | 770 | 936 |
| Gender |  |  |
| FEMALE | 780 | 984 |
| MALE | 1104 | 1408 |
| AJCC_Stage | 0 |  |
| Stage I | 300 | 240 |
| Stage II | 650 | 874 |
| Stage III | 638 | 822 |
| Stage IV | 230 | 444 |
| Un | 66 | 12 |
| histological_type |  |  |
| COAD | 832 | 0 |
| READ | 286 | 0 |
| STAD_Diffuse type | 130 | 390 |
| STAD_Mucinous type | 40 | 0 |
| STAD_Not Otherwise Specified | 392 | 0 |
| STAD_Papillary type | 14 | 0 |
| STAD_Signet ring type | 22 | 0 |
| STAD_Tubular type | 144 | 0 |
| Intestinal | 0 | 444 |
| Mixed | 0 | 60 |
| Un | 24 | 1498 |
| treatment_outcome_first_course | | |
| Complete remission/response | 970 | 0 |
| Partial remission/response | 40 | 0 |
| Progressive disease | 190 | 0 |
| Stable disease | 40 | 0 |
| Un | 644 | 0 |
