# Supplementary material for: A study on metabolic characteristics and metabolic markers of gastrointestinal tumors
Source: Cancer Biol Ther. 2023 Sep 13;24(1):2255369. doi: 10.1080/15384047.2023.2255369 (PMC10503448; doi:10.1080/15384047.2023.2255369)
Supplement: Supplemental Material [file KCBT_A_2255369_SM1060.zip › Supplementary material/Table S2.docx]

**Table S2: The list of 252 metabolism-related genes from KEGG and Reactome databases**

| ACADS |
| --- |
| ALDH1B1 |
| ACADL |
| ALDH2 |
| ACADM |
| CYP4A11 |
| ACADVL |
| ACAA2 |
| HADH |
| HADHB |
| HADHA |
| CYP4A22 |
| ADH7 |
| ADH6 |
| ADH1B |
| ADH1C |
| ADH4 |
| ECHS1 |
| ADH5 |
| ADH1A |
| ECI1 |
| ACOT2 |
| ACOT1 |
| ACOT7 |
| ACSS2 |
| GCK |
| PGK2 |
| PGK1 |
| PDHB |
| PDHA1 |
| PDHA2 |
| PGM2 |
| TPI1 |
| ACSS1 |
| FBP1 |
| HK2 |
| HK1 |
| HK3 |
| PGAM2 |
| PGAM1 |
| ALDOC |
| LDHAL6B |
| PKLR |
| LDHAL6A |
| ENO1 |
| PKM |
| PFKP |
| BPGM |
| PCK2 |
| PCK1 |
| ALDH3A1 |
| AKR1A1 |
| FBP2 |
| PFKM |
| PFKL |
| LDHC |
| GAPDH |
| ENO3 |
| ENO2 |
| LDHB |
| ALDOA |
| DLD |
| DLAT |
| ALDOB |
| G6PC2 |
| LDHA |
| G6PC |
| PGM1 |
| GPI |
| ME3 |
| HAGH |
| ME1 |
| ME2 |
| MDH2 |
| MDH1 |
| PC |
| GLO1 |
| AKR1B1 |
| ACOT12 |
| PFKFB4 |
| PFKFB3 |
| PFKFB2 |
| PFKFB1 |
| KHK |
| SORD |
| GALK1 |
| GLB1 |
| GALE |
| B4GALT1 |
| LALBA |
| GALT |
| UGP2 |
| B4GALT2 |
| GAA |
| UGT1A10 |
| UGT1A8 |
| UGT1A7 |
| UGT1A6 |
| UGT2B28 |
| UGT1A5 |
| UGDH |
| UGT2A1 |
| UGT1A9 |
| UGT2B11 |
| UGT2B10 |
| UGT2B7 |
| UGT2B4 |
| UGT2A3 |
| UGT1A4 |
| UGT1A1 |
| UGT2B17 |
| UGT1A3 |
| UGT2B15 |
| PRPS2 |
| PRPS1 |
| PAPSS1 |
| PAPSS2 |
| PRPS1L1 |
| ACY3 |
| GOT2 |
| GOT1 |
| GLYCTK |
| MAOB |
| MAOA |
| AOC2 |
| AOC3 |
| AHCY |
| MAT2B |
| MTR |
| MAT1A |
| MAT2A |
| AOC1 |
| ACY1 |
| COMT |
| GSTZ1 |
| OGDH |
| CYP1A1 |
| CYP1A2 |
| CYP1B1 |
| GGT7 |
| GGT5 |
| GGT1 |
| GGT6 |
| GSTP1 |
| GSTT2 |
| GSTT1 |
| PGD |
| GSTO1 |
| GSTA5 |
| MGST2 |
| MGST1 |
| MGST3 |
| GSTA3 |
| GSTM1 |
| GSTA4 |
| GSTM4 |
| GGCT |
| GSTM3 |
| GSTM2 |
| GSTM5 |
| GSTA1 |
| GSTA2 |
| GSS |
| GCLC |
| GSTK1 |
| IDH2 |
| OPLAH |
| GCLM |
| GSTO2 |
| G6PD |
| GYS1 |
| GYS2 |
| GUSB |
| PYGB |
| PYGM |
| PYGL |
| PGM2L1 |
| AGL |
| GBE1 |
| GNPDA2 |
| CYB5R3 |
| HEXB |
| HEXA |
| GNPDA1 |
| TKFC |
| CYP2E1 |
| CYP2J2 |
| PTGIS |
| CYP4F2 |
| CYP2C18 |
| PTGS1 |
| CYP2C9 |
| CYP2C19 |
| CYP2C8 |
| CYP2B6 |
| CBR3 |
| CYP2U1 |
| CYP4F3 |
| HPGDS |
| TBXAS1 |
| CYP3A5 |
| CYP3A4 |
| CYP3A43 |
| CYP3A7 |
| B4GALT6 |
| CS |
| PGP |
| ACO2 |
| MCEE |
| PCCB |
| PCCA |
| SUCLG1 |
| SUCLA2 |
| MMUT |
| SUCLG2 |
| ACSM1 |
| ACSM3 |
| ACSM4 |
| ACSM2A |
| ACSM5 |
| L2HGDH |
| NNT |
| NNMT |
| CYP26A1 |
| CYP26C1 |
| CYP2A13 |
| CYP26B1 |
| ALDH1A1 |
| CYP2A6 |
| CYP2A7 |
| SULT1E1 |
| SULT1A4 |
| SULT1A3 |
| CHST11 |
| SULT1A1 |
| SULT2B1 |
| BPNT1 |
| CHST13 |
| SULT1A2 |
| CHST12 |
| CYP2F1 |
| EPHX1 |
| CYP2S1 |
| CYP2D6 |
| FMO1 |
| FMO2 |
| FMO3 |
| CES2 |
| TPMT |
| NAT1 |
| CES1 |
| NAT2 |
