# Supplementary material for: A study on metabolic characteristics and metabolic markers of gastrointestinal tumors
Source: Cancer Biol Ther. 2023 Sep 13;24(1):2255369. doi: 10.1080/15384047.2023.2255369 (PMC10503448; doi:10.1080/15384047.2023.2255369)
Supplement: Supplemental Material [file KCBT_A_2255369_SM1060.zip › Supplementary material/Table S3.docx]

**Table S3: The differed expression of 151 genes in various MCluster subtypes**

| gName | MeanExpr | MedianExpr | VarExpr | W | P.value | FDR | MC1 | MC2 | MC3 | MC4 | MCluster |
| --- | --- | --- | --- | --- | --- | --- | --- | --- | --- | --- | --- |
| ALDH1A1 | 3870.299 | 1328.825555 | 12569.88 | 238.0624 | 2.50E-51 | 4.15E-49 | 13166.48 | 2057.052 | 2707.199 | 1464.151 | MC1 |
| CYB5R3 | 3822.154 | 3513.911112 | 1512.266 | 13.35825 | 0.003923 | 0.149057 | 4098.319 | 3758.304 | 3970.183 | 3507.852 | MC1 |
| OGDH | 3540.219 | 3372.93856 | 1197.005 | 13.03528 | 0.004561 | 0.164193 | 3763.247 | 3373.504 | 3547.312 | 3659.834 | MC1 |
| MDH1 | 2810.776 | 2687.018322 | 911.7546 | 20.83506 | 0.000114 | 0.00524 | 3009.116 | 2815.701 | 2838.684 | 2603.504 | MC1 |
| ADH5 | 2713.321 | 2549.831486 | 999.3846 | 3.533042 | 0.316502 | 1 | 2834.368 | 2724.935 | 2654.601 | 2679.323 | MC1 |
| MGST3 | 1973.198 | 1811.265 | 895.386 | 5.267601 | 0.153219 | 1 | 2212.196 | 1936.236 | 1943.128 | 1893.757 | MC1 |
| AOC3 | 945.007 | 393.6922538 | 1746.485 | 67.98666 | 1.15E-14 | 1.24E-12 | 2077.853 | 610.9237 | 732.8129 | 961.3914 | MC1 |
| HEXA | 1730.816 | 1620.493508 | 719.8763 | 35.08694 | 1.17E-07 | 7.82E-06 | 1948.467 | 1756.843 | 1740.879 | 1494.721 | MC1 |
| UGDH | 1532.328 | 1368.88783 | 779.8852 | 50.05123 | 7.79E-11 | 6.54E-09 | 1741.019 | 1607.567 | 1497.027 | 1277.46 | MC1 |
| PFKFB3 | 1469.433 | 1272.92 | 862.2538 | 49.48729 | 1.03E-10 | 8.42E-09 | 1714.625 | 1577.058 | 1387.844 | 1191.896 | MC1 |
| GAA | 1545.963 | 1338.953558 | 1025.558 | 4.093571 | 0.251536 | 1 | 1706.112 | 1501.941 | 1512.843 | 1546.829 | MC1 |
| PTGIS | 479.1103 | 120.9928915 | 1559.223 | 109.1899 | 1.64E-23 | 2.43E-21 | 1407.064 | 279.592 | 299.4687 | 364.0223 | MC1 |
| GCLC | 1162.296 | 1068.486226 | 502.5453 | 72.79583 | 1.08E-15 | 1.18E-13 | 1393.913 | 1110.21 | 1027.104 | 1267.073 | MC1 |
| PGM1 | 1328.554 | 1252.032413 | 625.1404 | 2.365294 | 0.500128 | 1 | 1363.728 | 1343.037 | 1286.171 | 1334.401 | MC1 |
| EPHX1 | 1058.777 | 863.2655569 | 770.0046 | 33.21909 | 2.90E-07 | 1.80E-05 | 1336.149 | 951.1928 | 991.386 | 1132.472 | MC1 |
| G6PD | 1201.092 | 994.201 | 776.9848 | 2.791521 | 0.424898 | 1 | 1251.035 | 1249.337 | 1154.27 | 1139.191 | MC1 |
| GSTM3 | 680.8718 | 401.1133241 | 801.5766 | 93.53639 | 3.81E-20 | 5.26E-18 | 1240.728 | 612.1046 | 573.5088 | 514.7254 | MC1 |
| FBP1 | 1007.961 | 825.1122264 | 806.62 | 2.545983 | 0.467038 | 1 | 1206.744 | 924.0873 | 1069.652 | 915.8952 | MC1 |
| AKR1B1 | 702.7547 | 534.7810975 | 612.0028 | 106.1871 | 7.26E-23 | 1.06E-20 | 1173.661 | 622.0261 | 646.4872 | 556.5468 | MC1 |
| CYP1B1 | 429.7873 | 85.27035042 | 922.464 | 122.4485 | 2.29E-26 | 3.57E-24 | 1149.225 | 291.0547 | 310.9829 | 281.8241 | MC1 |
| MTR | 898.2545 | 842.9615 | 364.2078 | 60.80475 | 3.96E-13 | 3.88E-11 | 1125.233 | 807.3265 | 861.9053 | 936.9948 | MC1 |
| GSTM4 | 875.8791 | 722.0155 | 647.7447 | 29.97278 | 1.40E-06 | 8.11E-05 | 1041.431 | 870.9328 | 879.925 | 747.1308 | MC1 |
| ALDH3A1 | 459.2349 | 152.7769202 | 1129.231 | 79.4862 | 3.96E-17 | 4.75E-15 | 1006.381 | 460.4217 | 324.7918 | 212.8594 | MC1 |
| GGT5 | 570.6502 | 386.8808543 | 601.9224 | 50.27047 | 7.00E-11 | 5.95E-09 | 895.5084 | 521.5324 | 520.3632 | 474.0672 | MC1 |
| MAOB | 395.6046 | 107.7235 | 763.2054 | 99.27296 | 2.23E-21 | 3.16E-19 | 878.4287 | 267.5218 | 345.8725 | 317.9399 | MC1 |
| GSTA1 | 294.7065 | 19.17345918 | 1262.035 | 96.99268 | 6.89E-21 | 9.71E-19 | 874.4943 | 139.1846 | 303.0807 | 107.1812 | MC1 |
| CES1 | 621.1071 | 114.1966532 | 1906.434 | 55.94512 | 4.32E-12 | 4.10E-10 | 852.5145 | 417.2992 | 620.9227 | 812.9147 | MC1 |
| PGM2L1 | 617.9323 | 519.5405562 | 421.3986 | 63.93696 | 8.47E-14 | 8.64E-12 | 834.6128 | 607.9778 | 570.5523 | 531.2161 | MC1 |
| PTGS1 | 476.4626 | 313.7258239 | 541.2531 | 85.17406 | 2.38E-18 | 3.02E-16 | 827.2008 | 413.0596 | 408.3934 | 411.0408 | MC1 |
| ADH1B | 316.6662 | 48.36055 | 888.9628 | 82.4923 | 8.96E-18 | 1.09E-15 | 812.8511 | 193.2357 | 203.8787 | 309.977 | MC1 |
| UGT1A10 | 357.1999 | 138.3235 | 788.3751 | 14.47235 | 0.002328 | 0.090787 | 710.2183 | 303.3968 | 238.1019 | 345.2548 | MC1 |
| UGT1A6 | 339.9711 | 129.3575 | 699.4366 | 6.190161 | 0.102716 | 1 | 670.5787 | 309.2185 | 241.2379 | 274.2225 | MC1 |
| CYP2C18 | 220.4729 | 80.60869873 | 369.9274 | 82.51845 | 8.85E-18 | 1.09E-15 | 503.8934 | 203.6037 | 133.63 | 149.7354 | MC1 |
| FMO2 | 168.6939 | 14.90864901 | 582.0991 | 163.8976 | 2.64E-35 | 4.25E-33 | 465.5137 | 164.7242 | 69.65751 | 80.90292 | MC1 |
| BPGM | 306.3472 | 276.1336317 | 152.3781 | 106.5652 | 6.02E-23 | 8.85E-21 | 402.0796 | 301.9668 | 290.2889 | 261.0273 | MC1 |
| ACOT1 | 341.0045 | 298.5587469 | 223.63 | 8.363049 | 0.039075 | 0.835534 | 381.3569 | 312.9509 | 360.4585 | 332.7522 | MC1 |
| GSTA4 | 258.9354 | 205.4899997 | 218.7107 | 55.4168 | 5.59E-12 | 5.26E-10 | 374.9749 | 237.2418 | 241.0379 | 232.0002 | MC1 |
| ADH4 | 109.058 | 6.4657 | 897.5038 | 42.42878 | 3.25E-09 | 2.41E-07 | 367.7479 | 92.63381 | 35.69164 | 37.94743 | MC1 |
| UGT1A1 | 105.32 | 4.6459 | 498.6669 | 95.57458 | 1.39E-20 | 1.93E-18 | 342.8364 | 77.15059 | 41.45292 | 59.18984 | MC1 |
| CHST12 | 278.7805 | 255.9585 | 132.4181 | 50.00276 | 7.98E-11 | 6.62E-09 | 338.2358 | 262.6672 | 283.0009 | 255.045 | MC1 |
| GNPDA2 | 259.4701 | 243.4592394 | 118.9707 | 33.54183 | 2.48E-07 | 1.56E-05 | 312.3178 | 241.9302 | 246.4136 | 268.3606 | MC1 |
| ADH6 | 189.4466 | 90.28007161 | 292.878 | 3.433111 | 0.329542 | 1 | 264.9178 | 181.7188 | 199.885 | 128.558 | MC1 |
| ACY3 | 230.8289 | 160.6348138 | 307.6077 | 9.139531 | 0.027492 | 0.659811 | 264.4072 | 263.7702 | 210.7551 | 171.9591 | MC1 |
| CYP2U1 | 180.6641 | 151.6525 | 119.7734 | 65.53267 | 3.86E-14 | 4.01E-12 | 256.5533 | 147.7537 | 167.749 | 199.3379 | MC1 |
| UGT2B7 | 121.7629 | 31.39936443 | 434.9683 | 3.600394 | 0.307973 | 1 | 214.7805 | 93.13997 | 136.2935 | 79.58607 | MC1 |
| UGT1A9 | 75.01369 | 11.8689 | 215.3408 | 46.40749 | 4.65E-10 | 3.72E-08 | 214.7798 | 57.79152 | 38.89745 | 46.96288 | MC1 |
| CHST11 | 137.0527 | 90.64330113 | 249.7076 | 77.8712 | 8.78E-17 | 1.02E-14 | 183.6791 | 132.2413 | 151.5908 | 87.92951 | MC1 |
| SULT1E1 | 99.08719 | 3.994079011 | 483.6613 | 91.62725 | 9.80E-20 | 1.32E-17 | 170.4973 | 95.79539 | 85.80257 | 67.19517 | MC1 |
| CYP2E1 | 53.57137 | 10.03339406 | 263.4037 | 83.80354 | 4.69E-18 | 5.86E-16 | 160.8223 | 39.29842 | 32.55321 | 24.41684 | MC1 |
| FMO3 | 55.1491 | 26.07048171 | 81.86929 | 166.7497 | 6.40E-36 | 1.04E-33 | 131.4478 | 37.90947 | 40.05641 | 47.68779 | MC1 |
| CYP2C9 | 43.40631 | 7.931497961 | 103.0683 | 113.4782 | 1.96E-24 | 2.96E-22 | 122.9586 | 33.60217 | 20.91965 | 30.20477 | MC1 |
| PYGM | 37.35448 | 7.888115734 | 135.7564 | 80.3356 | 2.60E-17 | 3.15E-15 | 107.8047 | 18.48988 | 23.20712 | 36.20617 | MC1 |
| GSTM5 | 44.37797 | 17.63470372 | 86.07791 | 112.6712 | 2.92E-24 | 4.38E-22 | 103.1435 | 32.26078 | 34 | 34.70518 | MC1 |
| CYP26B1 | 70.53338 | 40.04435186 | 102.2444 | 22.43545 | 5.29E-05 | 0.002647 | 101.4579 | 65.24703 | 73.71603 | 51.06292 | MC1 |
| GSTT2 | 70.32066 | 37.12582703 | 98.05828 | 12.5048 | 0.00584 | 0.198547 | 86.5851 | 69.29504 | 76.34268 | 50.62012 | MC1 |
| CHST13 | 44.16456 | 15.7457085 | 100.5839 | 9.914171 | 0.01931 | 0.50206 | 64.08822 | 32.45832 | 53.71886 | 36.20396 | MC1 |
| SULT1A2 | 39.50496 | 21.81247237 | 65.63626 | 4.453074 | 0.216514 | 1 | 46.49906 | 40.41502 | 36.91639 | 35.94866 | MC1 |
| HPGDS | 22.02201 | 10.4849 | 81.25789 | 127.091 | 2.29E-27 | 3.62E-25 | 43.7813 | 15.67848 | 23.20387 | 14.67837 | MC1 |
| PGAM2 | 21.35242 | 14.0007274 | 37.2198 | 31.11082 | 8.06E-07 | 4.83E-05 | 26.75884 | 17.69087 | 23.81763 | 20.26972 | MC1 |
| AOC2 | 11.74821 | 8.4196 | 13.88067 | 52.55914 | 2.28E-11 | 2.05E-09 | 16.23939 | 11.49332 | 11.86619 | 8.466452 | MC1 |
| CYP2C8 | 7.150568 | 2.582944714 | 19.57958 | 78.88146 | 5.33E-17 | 6.24E-15 | 15.07869 | 5.425995 | 6.936299 | 4.314651 | MC1 |
| ACSM1 | 8.866127 | 4.714915073 | 14.58165 | 19.2892 | 0.000238 | 0.01072 | 10.92104 | 8.326565 | 9.808699 | 6.873785 | MC1 |
| UGT1A3 | 4.307075 | 1.056074874 | 11.34267 | 89.08554 | 3.44E-19 | 4.54E-17 | 9.521977 | 2.570877 | 3.403598 | 4.643566 | MC1 |
| LDHAL6B | 2.65237 | 1.6019 | 3.530627 | 86.36444 | 1.32E-18 | 1.69E-16 | 4.797073 | 2.261813 | 2.017045 | 2.571094 | MC1 |
| PFKFB1 | 2.618598 | 1.686423605 | 4.4042 | 9.362248 | 0.024843 | 0.621072 | 4.141737 | 2.295354 | 2.198473 | 2.601088 | MC1 |
| ENO1 | 26016.91 | 23546.75 | 12281.65 | 209.1314 | 4.49E-45 | 7.40E-43 | 17395.39 | 31775.89 | 26295.5 | 21867.56 | MC2 |
| ALDOA | 26660.45 | 24552.1 | 12481.98 | 192.5319 | 1.73E-41 | 2.84E-39 | 16783.78 | 30865.56 | 28297.15 | 24437.31 | MC2 |
| LDHA | 17889.34 | 16559.71786 | 7991.505 | 179.3989 | 1.19E-38 | 1.94E-36 | 11894.23 | 21241.16 | 17818.9 | 16586.87 | MC2 |
| GSTP1 | 10797.24 | 9658.189241 | 6426.836 | 28.98505 | 2.26E-06 | 0.000124 | 11284.89 | 11436.01 | 11070.35 | 8838.363 | MC2 |
| HADHA | 4832.161 | 4703.69901 | 1286.089 | 14.92105 | 0.001885 | 0.077301 | 4517.119 | 4946.577 | 4919.832 | 4746.895 | MC2 |
| IDH2 | 4262.744 | 3753.87 | 2428.53 | 79.02368 | 4.97E-17 | 5.87E-15 | 3078.802 | 4729.112 | 4497.954 | 4010.034 | MC2 |
| PGAM1 | 4114.561 | 3894.403994 | 1548.646 | 140.8527 | 2.48E-30 | 3.96E-28 | 3458.201 | 4698.112 | 4259.723 | 3353.46 | MC2 |
| MAT2A | 4156.736 | 4017.098855 | 1224.223 | 10.21719 | 0.016807 | 0.487412 | 4093.637 | 4260.786 | 4022.348 | 4207.317 | MC2 |
| GSTK1 | 3508.95 | 3277.603549 | 1624.662 | 52.22331 | 2.68E-11 | 2.39E-09 | 2746.158 | 3688.922 | 3598.969 | 3656.572 | MC2 |
| ECHS1 | 3387.688 | 3111.131018 | 1455.578 | 69.66733 | 5.03E-15 | 5.48E-13 | 3031.013 | 3680.878 | 3582.11 | 2852.926 | MC2 |
| GOT2 | 3338.259 | 3234.455836 | 1159.804 | 137.7213 | 1.17E-29 | 1.86E-27 | 2420.613 | 3614.504 | 3416.587 | 3448.469 | MC2 |
| HK2 | 2844.837 | 2444.795 | 1982.794 | 96.51061 | 8.74E-21 | 1.22E-18 | 1890.685 | 3199.699 | 3021.335 | 2698.601 | MC2 |
| PGD | 2851.9 | 2631.402712 | 1378.941 | 42.46311 | 3.20E-09 | 2.40E-07 | 2715.733 | 3143.961 | 2823.721 | 2461.795 | MC2 |
| PFKP | 2637.977 | 2336.75 | 1475.849 | 63.40454 | 1.10E-13 | 1.11E-11 | 2270.838 | 3031.649 | 2679.367 | 2145.069 | MC2 |
| ACAA2 | 2365.474 | 2063.782808 | 1383.161 | 11.46197 | 0.009473 | 0.303136 | 2233.791 | 2595.319 | 2213.482 | 2263.784 | MC2 |
| GSTO1 | 2056.469 | 1859.39 | 1020.163 | 19.11587 | 0.000259 | 0.011384 | 1898.829 | 2161.808 | 2110.697 | 1910.224 | MC2 |
| GALE | 1885.336 | 1739.072258 | 962.6322 | 38.41137 | 2.31E-08 | 1.64E-06 | 1546.89 | 2058.793 | 1888.96 | 1830.027 | MC2 |
| ACOT7 | 1387.433 | 1242.871119 | 687.7256 | 66.74773 | 2.12E-14 | 2.25E-12 | 1161.781 | 1535.047 | 1472.095 | 1173.877 | MC2 |
| GOT1 | 1409.639 | 1278.007585 | 612.1123 | 52.05882 | 2.91E-11 | 2.56E-09 | 1216.673 | 1516.129 | 1489.475 | 1252.816 | MC2 |
| DLAT | 1332.91 | 1244.616163 | 506.2812 | 75.50655 | 2.82E-16 | 3.22E-14 | 1077.451 | 1454.876 | 1268.976 | 1403.093 | MC2 |
| SULT1A3 | 1286.39 | 1112.415 | 908.8258 | 21.08878 | 0.000101 | 0.004742 | 1047.697 | 1388.177 | 1238.057 | 1358.124 | MC2 |
| ME2 | 1086.272 | 989.3375 | 529.7597 | 18.77456 | 0.000304 | 0.013088 | 1082.274 | 1172.925 | 1055.675 | 973.3025 | MC2 |
| GGT6 | 990.7281 | 801.6384467 | 925.3642 | 74.06149 | 5.76E-16 | 6.45E-14 | 460.5087 | 1147.294 | 1078.91 | 998.6202 | MC2 |
| ACADS | 891.5775 | 728.8092199 | 590.0911 | 61.7079 | 2.54E-13 | 2.51E-11 | 630.8863 | 1005.535 | 966.5121 | 782.0197 | MC2 |
| PGM2 | 865.458 | 825.557515 | 313.9629 | 29.7399 | 1.57E-06 | 8.92E-05 | 835.6125 | 937.512 | 820.8345 | 820.1272 | MC2 |
| GSTT1 | 770.9199 | 640.6065 | 723.5739 | 3.625239 | 0.304879 | 1 | 794.4099 | 806.6173 | 744.6944 | 723.8142 | MC2 |
| PYGL | 712.5305 | 501.6352747 | 664.3276 | 26.20367 | 8.65E-06 | 0.000458 | 509.6767 | 799.3931 | 784.773 | 610.6877 | MC2 |
| ME1 | 628.2834 | 511.5460725 | 459.6346 | 13.29749 | 0.004036 | 0.149314 | 621.84 | 697.142 | 548.6941 | 620.2095 | MC2 |
| ENO2 | 599.804 | 431.1315 | 554.3541 | 34.25725 | 1.75E-07 | 1.15E-05 | 622.9138 | 654.6557 | 631.7528 | 434.4093 | MC2 |
| PGP | 546.9017 | 478.8484127 | 324.6447 | 53.17759 | 1.68E-11 | 1.53E-09 | 419.8272 | 589.2914 | 576.5134 | 527.6802 | MC2 |
| GBE1 | 470.389 | 444.9545385 | 189.6083 | 8.426131 | 0.037979 | 0.835534 | 448.0594 | 488.4967 | 477.1962 | 445.043 | MC2 |
| GSTZ1 | 302.0385 | 260.3328312 | 175.0979 | 66.15089 | 2.85E-14 | 2.99E-12 | 290.559 | 335.175 | 320.1508 | 224.1204 | MC2 |
| L2HGDH | 311.9167 | 299.0983485 | 135.866 | 40.42289 | 8.67E-09 | 6.33E-07 | 259.7033 | 334.6039 | 314.7601 | 307.6331 | MC2 |
| PFKFB4 | 262.1626 | 193.9363062 | 229.5288 | 114.2895 | 1.31E-24 | 1.99E-22 | 147.1602 | 301.2913 | 297.4259 | 231.2417 | MC2 |
| NAT1 | 218.8588 | 168.6710059 | 173.3221 | 29.63232 | 1.65E-06 | 9.23E-05 | 238.6691 | 241.5088 | 204.8005 | 181.3619 | MC2 |
| NAT2 | 87.45633 | 48.74900882 | 109.8715 | 83.04038 | 6.83E-18 | 8.48E-16 | 33.84093 | 107.4504 | 89.32456 | 90.65736 | MC2 |
| GAPDH | 67227.91 | 59477.26395 | 35476.47 | 432.776 | 1.76E-93 | 2.97E-91 | 38644.19 | 67141.79 | 96832.96 | 47832.63 | MC3 |
| TPI1 | 11292.24 | 10359.96243 | 5124.959 | 241.8782 | 3.74E-52 | 6.24E-50 | 7871.739 | 12076.7 | 13843.63 | 8922.791 | MC3 |
| AHCY | 8966.593 | 7517.252624 | 5814.189 | 105.9181 | 8.29E-23 | 1.20E-20 | 5876.532 | 8474.801 | 10388.3 | 10305.54 | MC3 |
| GPI | 9200.022 | 8778.605 | 3524.987 | 118.3414 | 1.76E-25 | 2.69E-23 | 7393.269 | 9782.915 | 10246.5 | 8068.352 | MC3 |
| LDHB | 7105.124 | 6493.811255 | 5736.754 | 43.24468 | 2.18E-09 | 1.68E-07 | 5673.334 | 6901.163 | 8526.687 | 6589.923 | MC3 |
| MDH2 | 6517.424 | 6174.691288 | 2511.94 | 99.84353 | 1.68E-21 | 2.40E-19 | 5068.309 | 6586.128 | 7162.197 | 6624.246 | MC3 |
| ALDH2 | 6421.242 | 6148.510499 | 2787.969 | 10.15614 | 0.017284 | 0.487412 | 6230.238 | 6322.574 | 6916.375 | 6047.291 | MC3 |
| CS | 4591.841 | 4446.468982 | 1359.697 | 123.5905 | 1.30E-26 | 2.04E-24 | 3714.149 | 4670.152 | 5166.749 | 4324.978 | MC3 |
| ACADVL | 4673.027 | 4274.204112 | 1916.754 | 38.01069 | 2.81E-08 | 1.94E-06 | 4601.639 | 4890.171 | 4913.365 | 3985.304 | MC3 |
| ACO2 | 3491.927 | 3315.741723 | 1267.794 | 5.330016 | 0.149167 | 1 | 3322.315 | 3488.034 | 3649.264 | 3409.314 | MC3 |
| AKR1A1 | 2727.418 | 2575.451106 | 998.3271 | 76.74165 | 1.53E-16 | 1.76E-14 | 2646.454 | 2765.957 | 3056.339 | 2250.245 | MC3 |
| COMT | 2328.479 | 2047.425 | 1460.491 | 46.81381 | 3.81E-10 | 3.08E-08 | 1914.09 | 2289.513 | 2774.465 | 2092.892 | MC3 |
| SUCLG1 | 2448.546 | 2290.072753 | 1066.202 | 87.93809 | 6.07E-19 | 7.83E-17 | 1832.573 | 2493.098 | 2658.595 | 2557.234 | MC3 |
| GUSB | 2013.031 | 1813.74 | 938.9091 | 33.86405 | 2.12E-07 | 1.38E-05 | 2158.262 | 1864.263 | 2221.099 | 1874.029 | MC3 |
| PCK2 | 1729.906 | 1562.467153 | 1031.174 | 111.4922 | 5.24E-24 | 7.81E-22 | 1060.259 | 1748.244 | 1990.188 | 1857.927 | MC3 |
| SORD | 1431.454 | 1297.36 | 792.4458 | 92.94719 | 5.10E-20 | 6.98E-18 | 994.0814 | 1564.275 | 1580.716 | 1321.696 | MC3 |
| PCCB | 1316.825 | 1210.642889 | 510.5482 | 51.99464 | 3.00E-11 | 2.61E-09 | 1246.331 | 1365.7 | 1416.739 | 1139.898 | MC3 |
| PFKM | 1204.352 | 1150.572993 | 510.0816 | 38.30442 | 2.44E-08 | 1.71E-06 | 1083.183 | 1162.967 | 1351.094 | 1167.448 | MC3 |
| GYS1 | 1249.616 | 1209.356638 | 391.0966 | 39.37758 | 1.44E-08 | 1.04E-06 | 1169.882 | 1273.342 | 1338.188 | 1142.709 | MC3 |
| PCCA | 926.5681 | 490.9636516 | 1484.479 | 74.92799 | 3.75E-16 | 4.24E-14 | 437.7735 | 861.2054 | 1306.147 | 894.0856 | MC3 |
| PDHB | 1203.938 | 1163.883721 | 361.25 | 21.74632 | 7.37E-05 | 0.003536 | 1100.132 | 1208.197 | 1248.913 | 1214.546 | MC3 |
| B4GALT2 | 1084.717 | 1023.269268 | 401.9394 | 50.7768 | 5.46E-11 | 4.69E-09 | 930.3681 | 1121.473 | 1171.3 | 1016.139 | MC3 |
| ALDOB | 941.204 | 271.8320009 | 2150.548 | 11.55623 | 0.009069 | 0.299269 | 852.0255 | 882.2888 | 1038.858 | 981.3717 | MC3 |
| PRPS1 | 826.6228 | 769.7844388 | 358.509 | 2.069193 | 0.55817 | 1 | 828.6399 | 817.5705 | 853.0694 | 803.8802 | MC3 |
| ALDOC | 638.0527 | 503.5568121 | 526.6897 | 91.36519 | 1.12E-19 | 1.49E-17 | 341.9885 | 698.0154 | 704.47 | 668.5649 | MC3 |
| HAGH | 581.9989 | 541.5966946 | 229.315 | 23.05361 | 3.94E-05 | 0.002007 | 521.3375 | 571.4592 | 616.3742 | 600.6665 | MC3 |
| SULT2B1 | 408.8843 | 285.7616517 | 473.1062 | 122.3456 | 2.41E-26 | 3.74E-24 | 167.6682 | 467.8756 | 482.8683 | 386.6013 | MC3 |
| GALT | 400.5341 | 366.8537232 | 175.2073 | 18.1075 | 0.000418 | 0.017554 | 386.3247 | 384.4161 | 433.4217 | 394.5641 | MC3 |
| GSTO2 | 372.9349 | 333.4568921 | 244.4666 | 36.22224 | 6.72E-08 | 4.57E-06 | 322.4904 | 380.0778 | 424.7524 | 325.8562 | MC3 |
| CBR3 | 114.3489 | 82.44988843 | 96.98926 | 30.60301 | 1.03E-06 | 6.08E-05 | 107.3076 | 124.3788 | 127.723 | 82.3178 | MC3 |
| HK3 | 89.55148 | 52.75994043 | 114.3589 | 14.63519 | 0.002156 | 0.08626 | 69.65926 | 93.79108 | 104.5459 | 76.14289 | MC3 |
| CYP3A4 | 59.91201 | 4.490351117 | 346.3064 | 8.627782 | 0.034672 | 0.797451 | 71.46983 | 41.98349 | 73.5183 | 64.31323 | MC3 |
| ENO3 | 46.65189 | 35.47655797 | 45.27547 | 11.2393 | 0.0105 | 0.325495 | 48.18762 | 46.78028 | 52.45342 | 36.88847 | MC3 |
| GCK | 4.417863 | 2.112063628 | 17.74403 | 8.186329 | 0.042314 | 0.846276 | 5.554008 | 3.59497 | 5.598643 | 3.340692 | MC3 |
| PYGB | 10771.8 | 7569.618254 | 9645.625 | 249.6782 | 7.68E-54 | 1.29E-51 | 6527.143 | 8319.085 | 8932.733 | 21317.02 | MC4 |
| PGK1 | 12845.35 | 11178.41805 | 6293.021 | 103.6851 | 2.51E-22 | 3.61E-20 | 9018.126 | 13882.91 | 12793.73 | 14057.8 | MC4 |
| CES2 | 4768.856 | 3403.526944 | 4485.643 | 59.04586 | 9.40E-13 | 9.12E-11 | 3855.33 | 4841.006 | 3913.331 | 6588.693 | MC4 |
| CYP2S1 | 3609.025 | 3182.614721 | 2351.707 | 12.99461 | 0.004648 | 0.164193 | 3350.97 | 3508.293 | 3708.531 | 3858.469 | MC4 |
| HEXB | 3418.816 | 3274.248453 | 1190.174 | 25.43615 | 1.25E-05 | 0.000651 | 3099.542 | 3540.197 | 3300.053 | 3619.449 | MC4 |
| GLO1 | 3310.953 | 2981.285 | 1735.467 | 62.2927 | 1.90E-13 | 1.90E-11 | 2636.919 | 3516.284 | 3211.937 | 3611.435 | MC4 |
| ALDH1B1 | 3067.23 | 2425.943069 | 2394.479 | 57.78868 | 1.74E-12 | 1.67E-10 | 2512.506 | 2839.561 | 3399.526 | 3454.579 | MC4 |
| ACSS2 | 2461.563 | 2281.17 | 1335.279 | 88.78684 | 3.99E-19 | 5.23E-17 | 1726.62 | 2435.629 | 2545.491 | 2975.731 | MC4 |
| PDHA1 | 2582.102 | 2299.43262 | 1189.493 | 55.28844 | 5.96E-12 | 5.54E-10 | 2078.439 | 2585.809 | 2612.003 | 2934.337 | MC4 |
| DLD | 2345.96 | 2249.475856 | 824.9534 | 22.20429 | 5.91E-05 | 0.002898 | 2213.391 | 2370.162 | 2292.282 | 2483.866 | MC4 |
| ACSS1 | 1781.654 | 1504.472671 | 1231.358 | 92.44898 | 6.52E-20 | 8.87E-18 | 1910.893 | 1448.776 | 1652.462 | 2477.8 | MC4 |
| GSS | 2020.971 | 1828.51 | 1024.993 | 120.0769 | 7.43E-26 | 1.14E-23 | 1371.683 | 2027.692 | 2080.009 | 2442.14 | MC4 |
| GLB1 | 2143.963 | 2065.648859 | 879.5912 | 79.09121 | 4.81E-17 | 5.72E-15 | 1749.72 | 2133.407 | 2235.514 | 2347.006 | MC4 |
| UGP2 | 2138.846 | 2063.945442 | 715.45 | 27.64575 | 4.31E-06 | 0.000233 | 2168.142 | 2207.151 | 1922.049 | 2299.605 | MC4 |
| PRPS2 | 1765.844 | 1645.205742 | 800.9684 | 67.49483 | 1.47E-14 | 1.57E-12 | 1386.191 | 1784.961 | 1843.728 | 1922.03 | MC4 |
| MAT2B | 1842.443 | 1760.078514 | 638.7584 | 0.709006 | 0.871084 | 1 | 1787.376 | 1858.976 | 1829.169 | 1874.857 | MC4 |
| TPMT | 1439.359 | 1328.216528 | 679.533 | 64.35635 | 6.89E-14 | 7.09E-12 | 1152.047 | 1556.332 | 1322.349 | 1620.116 | MC4 |
| GNPDA1 | 1307.794 | 1217.145681 | 504.8958 | 33.65511 | 2.34E-07 | 1.50E-05 | 1153.639 | 1315.045 | 1286.72 | 1447.559 | MC4 |
| SUCLA2 | 1177.395 | 1043.700095 | 568.0153 | 43.13434 | 2.30E-09 | 1.75E-07 | 1046.207 | 1179.672 | 1110.238 | 1373.956 | MC4 |
| PCK1 | 800.3751 | 297.7091006 | 1571.724 | 73.49579 | 7.61E-16 | 8.45E-14 | 353.8444 | 733.8358 | 851.9864 | 1205.616 | MC4 |
| OPLAH | 857.147 | 700.5615169 | 608.6963 | 4.911568 | 0.178388 | 1 | 814.2853 | 859.9941 | 853.4594 | 891.3665 | MC4 |
| GGT7 | 485.6755 | 402.659 | 372.1704 | 88.69682 | 4.17E-19 | 5.42E-17 | 431.4253 | 405.7221 | 463.7511 | 707.9043 | MC4 |
| CYP2B6 | 491.1388 | 261.7145253 | 632.0901 | 54.61184 | 8.31E-12 | 7.64E-10 | 358.6528 | 480.336 | 489.6493 | 618.9162 | MC4 |
| TBXAS1 | 531.0087 | 387.099841 | 470.4941 | 31.68068 | 6.11E-07 | 3.73E-05 | 363.3295 | 505.4335 | 598.9939 | 614.7242 | MC4 |
| GGT1 | 545.0372 | 346.1971751 | 604.1209 | 2.859931 | 0.413729 | 1 | 534.7864 | 541.4302 | 535.1528 | 574.0169 | MC4 |
| SULT1A1 | 417.0616 | 306.588 | 416.9259 | 5.846464 | 0.119324 | 1 | 360.1047 | 442.2685 | 386.7369 | 459.3708 | MC4 |
| ACSM3 | 411.426 | 291.8969681 | 446.159 | 10.03422 | 0.018277 | 0.493491 | 381.9533 | 419.0111 | 404.7594 | 430.48 | MC4 |
| PC | 333.8848 | 284.2858973 | 214.6103 | 10.98807 | 0.011791 | 0.353717 | 336.2462 | 333.1681 | 318.2484 | 355.6971 | MC4 |
| CYP4F3 | 242.6275 | 180.6971005 | 253.4819 | 45.88697 | 5.99E-10 | 4.74E-08 | 269.8002 | 204.7505 | 228.458 | 311.1328 | MC4 |
| CYP4F2 | 143.5343 | 44.34656971 | 277.0313 | 91.21126 | 1.20E-19 | 1.60E-17 | 62.99021 | 145.0406 | 119.1263 | 239.9421 | MC4 |
| UGT2B15 | 213.5306 | 55.17905 | 469.9292 | 3.782954 | 0.285875 | 1 | 208.6489 | 214.8758 | 202.2513 | 231.0815 | MC4 |
| B4GALT6 | 186.9538 | 154.7780693 | 154.4607 | 8.064122 | 0.044705 | 0.849393 | 176.0977 | 182.4177 | 189.5513 | 200.2717 | MC4 |
| PKLR | 64.61146 | 8.128525636 | 150.5823 | 83.89098 | 4.49E-18 | 5.66E-16 | 27.00105 | 48.33241 | 82.20039 | 99.49958 | MC4 |
| CYP3A7 | 9.484851 | 4.313742347 | 22.27249 | 44.93338 | 9.56E-10 | 7.46E-08 | 7.260931 | 6.627335 | 11.76775 | 13.26701 | MC4 |
