# Supplementary material for: A study on metabolic characteristics and metabolic markers of gastrointestinal tumors
Source: Cancer Biol Ther. 2023 Sep 13;24(1):2255369. doi: 10.1080/15384047.2023.2255369 (PMC10503448; doi:10.1080/15384047.2023.2255369)
Supplement: Supplemental Material [file KCBT_A_2255369_SM1060.zip › Supplementary material/Table S4.docx]

**Table S4: Comparison of the distribution of samples with various clinical characteristics in Mclusters**

|  | TCGA | MC1 | MC2 | MC3 | MC4 | Chisq-test p |
| --- | --- | --- | --- | --- | --- | --- |
| Event |  |  |  |  |  |  |
| Alive | 676 | 81(12.0%) | 252(37.3%) | 190(28.1%) | 153(22.6%) |  |
| Dead | 266 | 69(25.9%) | 94(35.3%) | 72(27.1%) | 31(11.7%) |  |
| Age |  |  |  |  |  | **0.0013**** |
| 0~50 | 103 | 11(10.7%) | 41(39.8%) | 36(35.0%) | 15(14.6%) |  |
| 50~60 | 182 | 44(24.2%) | 58(31.9%) | 36(19.8%) | 44(24.2%) |  |
| 60~70 | 272 | 50(18.4%) | 98(36.0%) | 74(27.2%) | 50(18.4%) |  |
| 70~100 | 385 | 45(11.7%) | 149(38.7%) | 116(30.1%) | 75(19.5%) |  |
| Gender |  |  |  |  |  | 0.24892 |
| FEMALE | 390 | 51(13.1%) | 149(38.2%) | 113(29.0%) | 77(19.7%) |  |
| MALE | 552 | 99(17.9%) | 197(35.7%) | 149(27.0%) | 107(19.4%) |  |
| AJCC_Stage |  |  |  |  |  | **0.04982*** |
| Stage I | 150 | 17(11.3%) | 60(40.0%) | 46(30.7%) | 27(18.0%) |  |
| Stage II | 325 | 45(13.8%) | 113(34.8%) | 107(32.9%) | 60(18.5%) |  |
| Stage III | 319 | 65(20.4%) | 121(37.9%) | 70(21.9%) | 63(19.7%) |  |
| Stage IV | 115 | 15(13.0%) | 44(38.3%) | 30(26.1%) | 26(22.6%) |  |
| Un | 33 | 8(24.2%) | 8(24.2%) | 9(27.3%) | 8(24.2%) |  |
| histological_type |  |  |  |  |  | **<1e-5***** |
| COAD | 416 | 8(1.9%) | 172(41.3%) | 137(32.9%) | 99(23.8%) |  |
| READ | 143 | 3(2.1%) | 51(35.7%) | 46(32.2%) | 43(30.1%) |  |
| STAD_Diffuse type | 65 | 29(44.6%) | 17(26.2%) | 16(24.6%) | 3(4.6%) |  |
| STAD_Mucinous type | 20 | 4(20.0%) | 7(35.0%) | 7(35.0%) | 2(10.0%) |  |
| STAD_Not Otherwise Specified | 196 | 68(34.7%) | 72(36.7%) | 37(18.9%) | 19(9.7%) |  |
| STAD_Papillary type | 7 | 1(14.3%) | 4(57.1%) | 1(14.3%) | 1(14.3%) |  |
| STAD_Signet ring type | 11 | 6(54.5%) | 3(27.3%) | 0(0.0%) | 2(18.2%) |  |
| STAD_Tubular type | 72 | 29(40.3%) | 17(23.6%) | 12(16.7%) | 14(19.4%) |  |
| Un | 12 | 2(16.7%) | 3(25.0%) | 6(50.0%) | 1(8.3%) |  |
| treatment_outcome_first_course |  |  |  |  |  | 0.61442 |
| Complete response | 485 | 96(19.8%) | 181(37.3%) | 119(24.5%) | 89(18.4%) |  |
| Partial response | 20 | 3(15.0%) | 8(40.0%) | 5(25.0%) | 4(20.0%) |  |
| Progressive disease | 95 | 27(28.4%) | 34(35.8%) | 21(22.1%) | 13(13.7%) |  |
| Stable disease | 20 | 7(35.0%) | 6(30.0%) | 3(15.0%) | 4(20.0%) |  |
| Un | 322 | 17(5.3%) | 117(36.3%) | 114(35.4%) | 74(23.0%) |  |

* * * represented p <1×e-3, * * represented p <0.01 and * represented p <0.05
