# Supplementary material for: A study on metabolic characteristics and metabolic markers of gastrointestinal tumors
Source: Cancer Biol Ther. 2023 Sep 13;24(1):2255369. doi: 10.1080/15384047.2023.2255369 (PMC10503448; doi:10.1080/15384047.2023.2255369)
Supplement: Supplemental Material [file KCBT_A_2255369_SM1060.zip › Supplementary material/Table S5.docx]

**Table S5: Analysis of the scores of four metabolism-related gene signatures in Pan-cancer**

|  | gSig1.Score | gSig2.Score | gSig3.Score | gSig4.Score |
| --- | --- | --- | --- | --- |
| ACC | 11.36698 | 12.02113 | 12.8411 | 11.10232 |
| BLCA | 9.890478 | 12.13283 | 12.30665 | 10.37183 |
| BRCA | 9.905174 | 11.60746 | 11.68468 | 10.32961 |
| CESC | 9.587537 | 12.30826 | 12.1242 | 10.54382 |
| CHOL | 10.1688 | 11.66157 | 12.04756 | 10.62867 |
| COAD | 9.152879 | 11.98939 | 12.24299 | 11.12556 |
| DLBC | 9.636573 | 12.3059 | 12.54043 | 10.30531 |
| ESCA | 9.784211 | 11.9033 | 11.75312 | 10.64795 |
| GBM | 10.04591 | 11.77959 | 12.33258 | 10.54217 |
| HNSC | 9.83214 | 12.55662 | 12.4097 | 10.71175 |
| KICH | 10.31384 | 12.25106 | 12.49974 | 11.30975 |
| KIRC | 10.62856 | 12.57428 | 12.53389 | 10.97342 |
| KIRP | 10.7026 | 12.37462 | 12.5921 | 11.18639 |
| LGG | 9.914892 | 11.33933 | 12.13843 | 10.20891 |
| LIHC | 12.68867 | 12.02615 | 13.20517 | 11.79265 |
| LUAD | 10.1506 | 12.02637 | 11.90118 | 10.63535 |
| LUSC | 10.38667 | 12.31256 | 12.5586 | 10.90682 |
| MESO | 10.4694 | 12.03113 | 12.10775 | 10.43155 |
| OV | 9.745211 | 12.08312 | 12.45642 | 10.73895 |
| PAAD | 9.895486 | 11.65192 | 11.69696 | 10.41077 |
| PCPG | 10.09992 | 11.8066 | 12.04395 | 10.22084 |
| PRAD | 9.908823 | 11.12039 | 11.6649 | 10.66515 |
| READ | 9.144988 | 11.89675 | 12.21625 | 11.16728 |
| SARC | 10.31191 | 11.6205 | 11.82907 | 10.59857 |
| SKCM | 10.20293 | 12.33484 | 12.65739 | 10.86099 |
| STAD | 9.666574 | 11.66293 | 11.79607 | 10.6994 |
| TGCT | 9.565743 | 11.90288 | 12.87317 | 10.57376 |
| THCA | 9.840236 | 11.39504 | 11.58922 | 10.59796 |
| THYM | 9.863905 | 11.50249 | 11.92904 | 10.34152 |
| UCEC | 9.848346 | 11.97611 | 12.3182 | 10.27207 |
| UCS | 9.737521 | 11.95615 | 12.49874 | 10.48672 |
| UVM | 10.25934 | 12.40821 | 12.90174 | 10.85108 |
