# Supplementary figures and images for: A study on metabolic characteristics and metabolic markers of gastrointestinal tumors
Source: Cancer Biol Ther. 2023 Sep 13;24(1):2255369. doi: 10.1080/15384047.2023.2255369 (PMC10503448; doi:10.1080/15384047.2023.2255369)

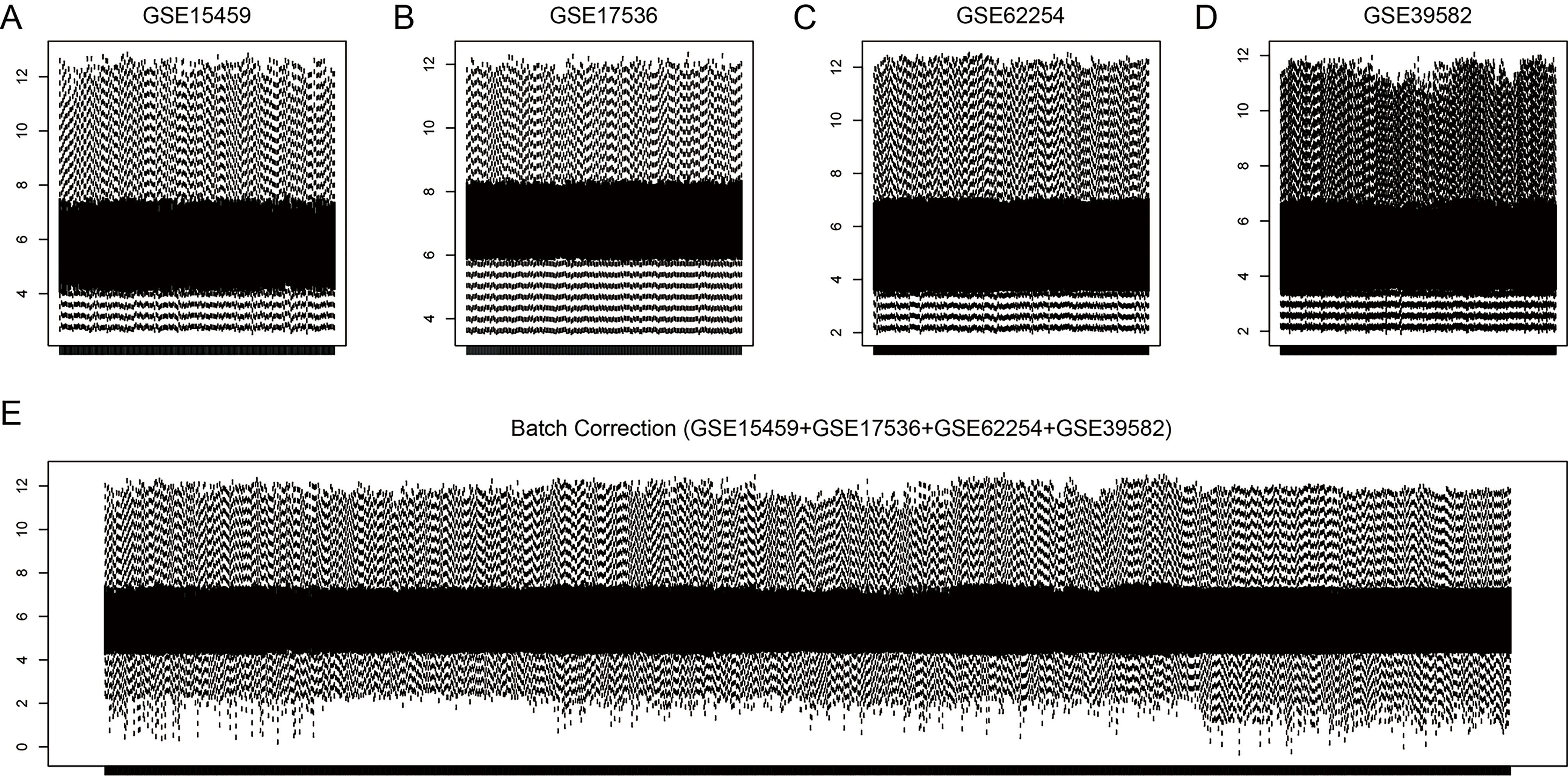

Supplement: Supplemental Material [file KCBT_A_2255369_SM1060.zip › Supplementary material/S1_Fig.png]

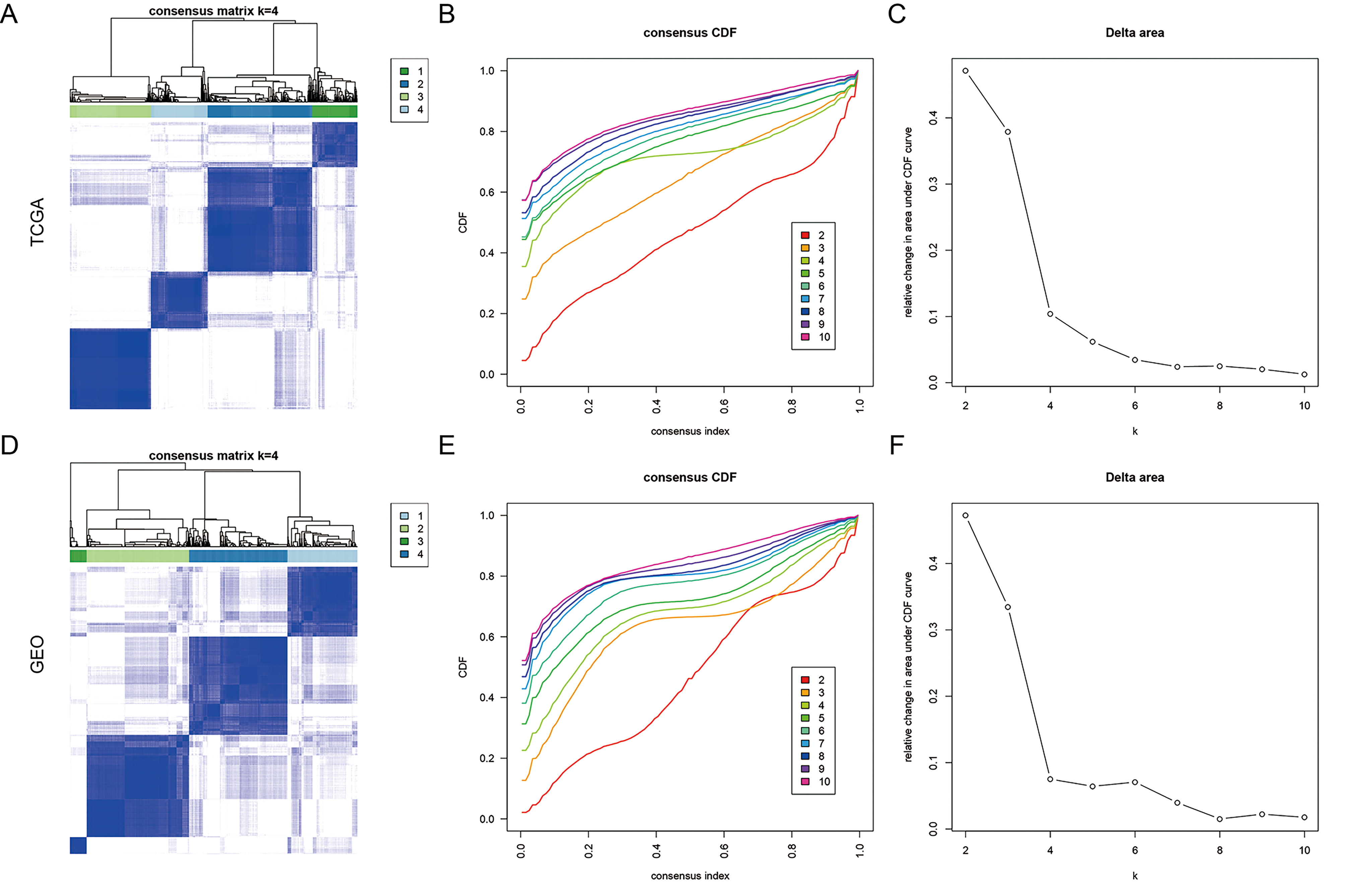

Supplement: Supplemental Material [file KCBT_A_2255369_SM1060.zip › Supplementary material/S2_Fig.png]

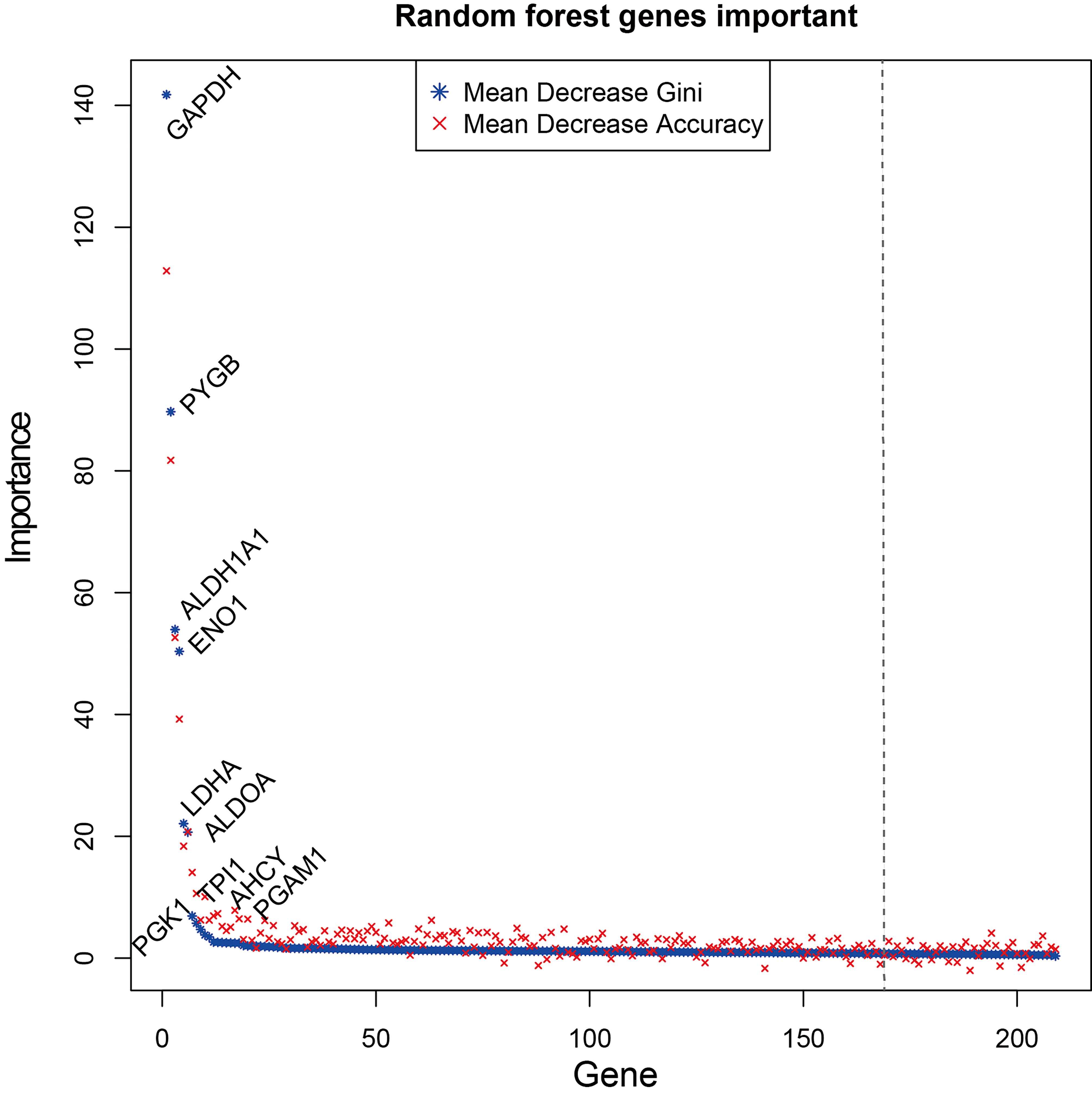

Supplement: Supplemental Material [file KCBT_A_2255369_SM1060.zip › Supplementary material/S3_Fig.png]

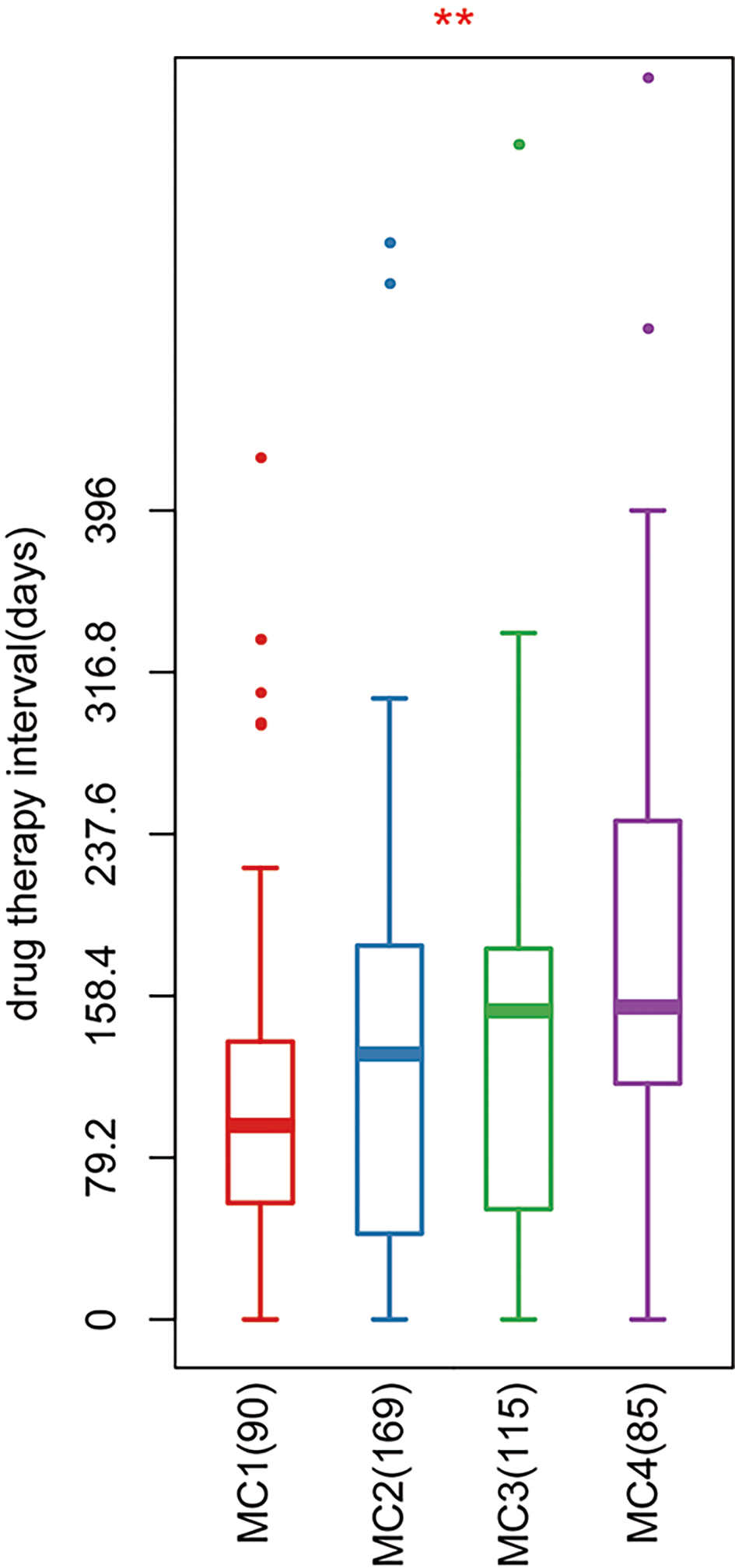

Supplement: Supplemental Material [file KCBT_A_2255369_SM1060.zip › Supplementary material/S4_Fig.png]

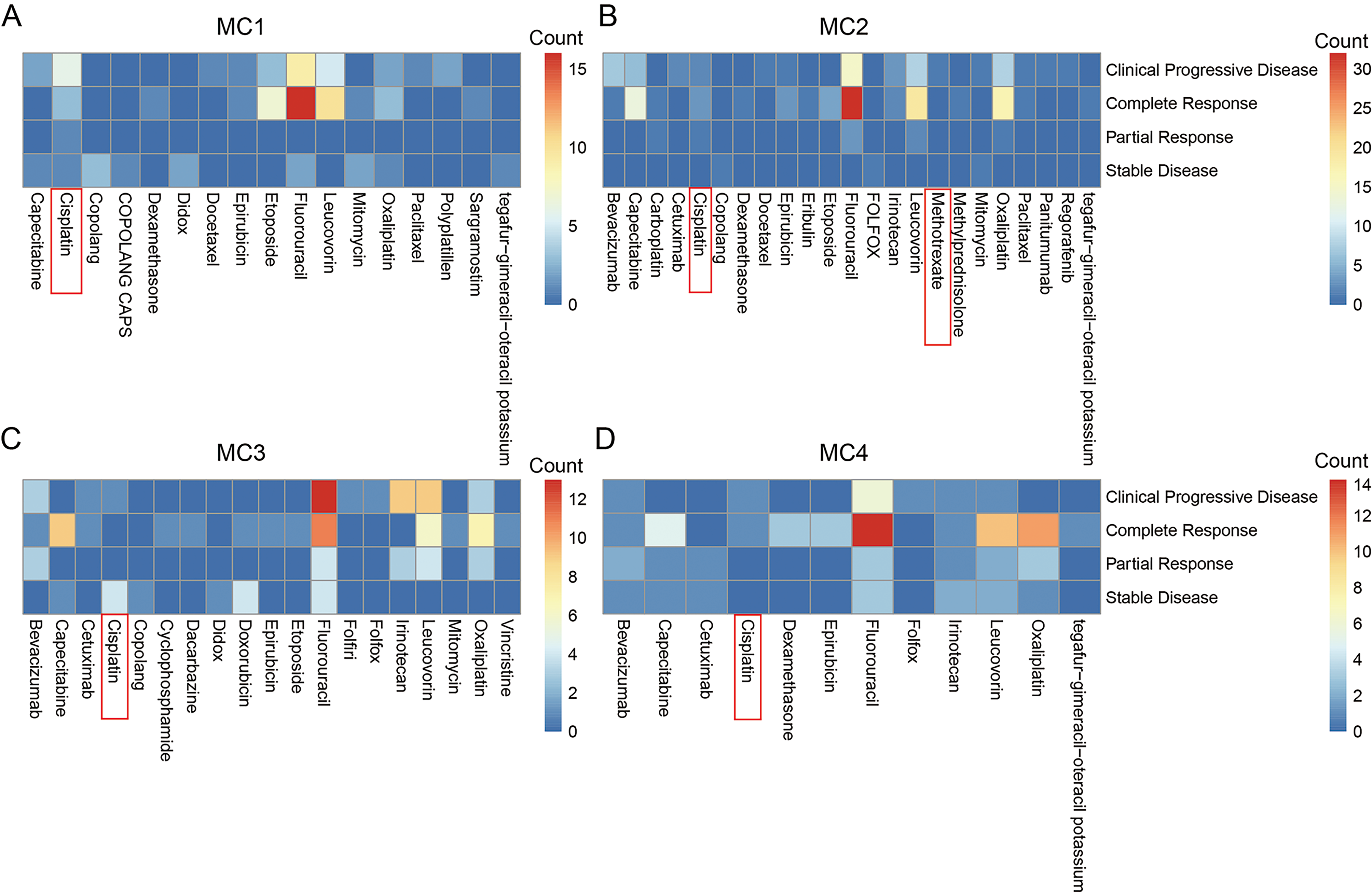

Supplement: Supplemental Material [file KCBT_A_2255369_SM1060.zip › Supplementary material/S5_Fig.png]

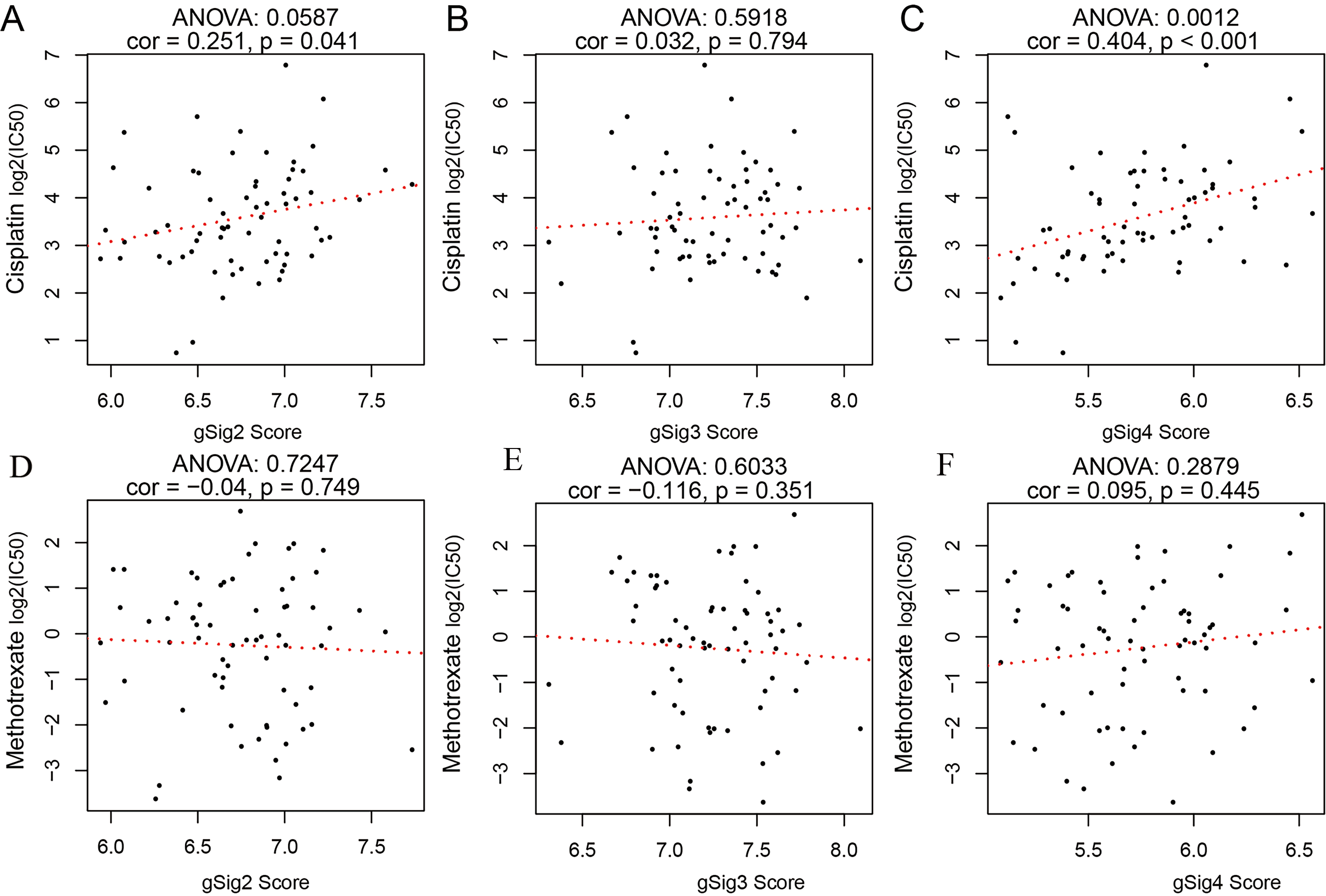

Supplement: Supplemental Material [file KCBT_A_2255369_SM1060.zip › Supplementary material/S6_Fig.png]

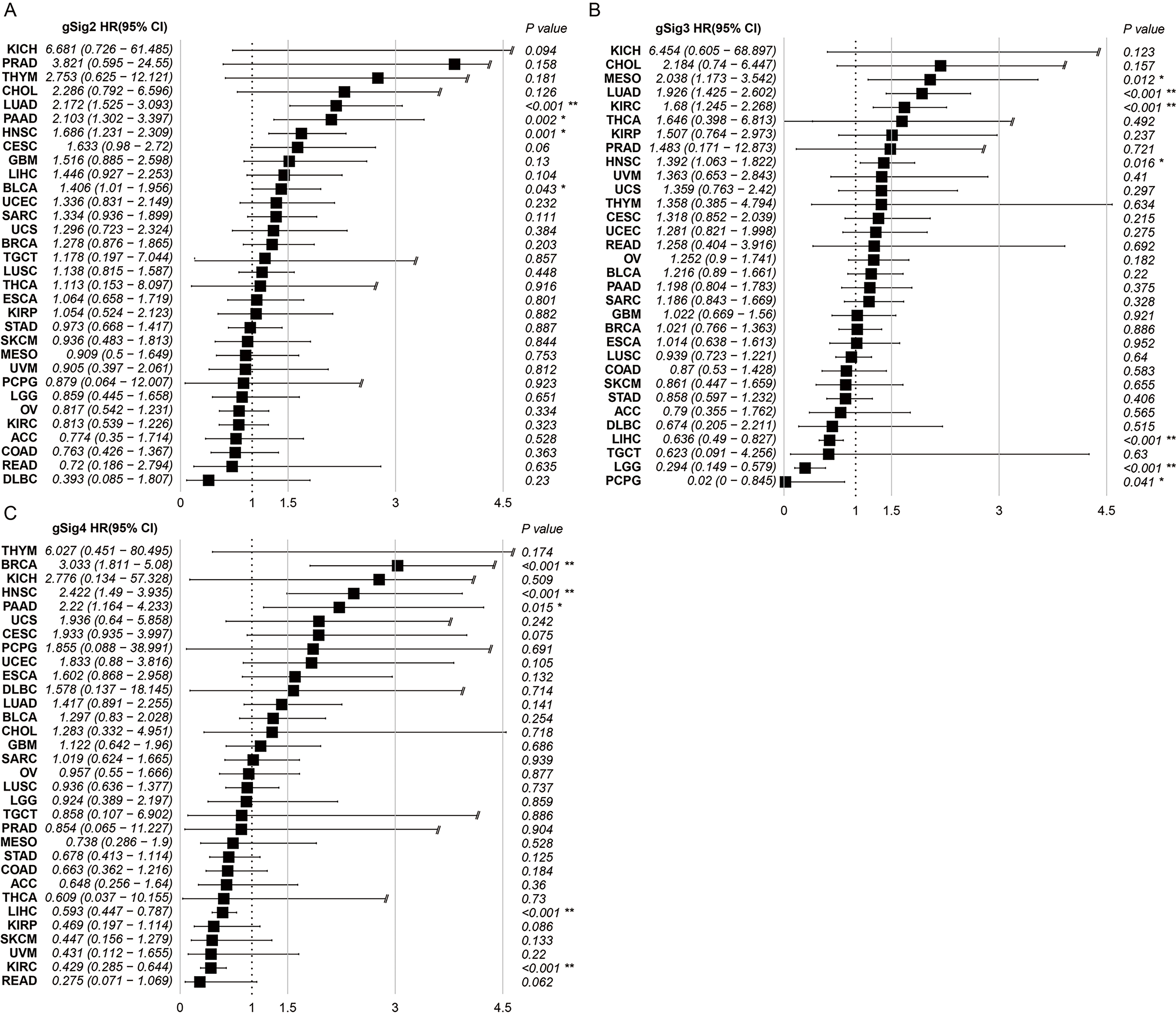

Supplement: Supplemental Material [file KCBT_A_2255369_SM1060.zip › Supplementary material/S7_Fig.png]

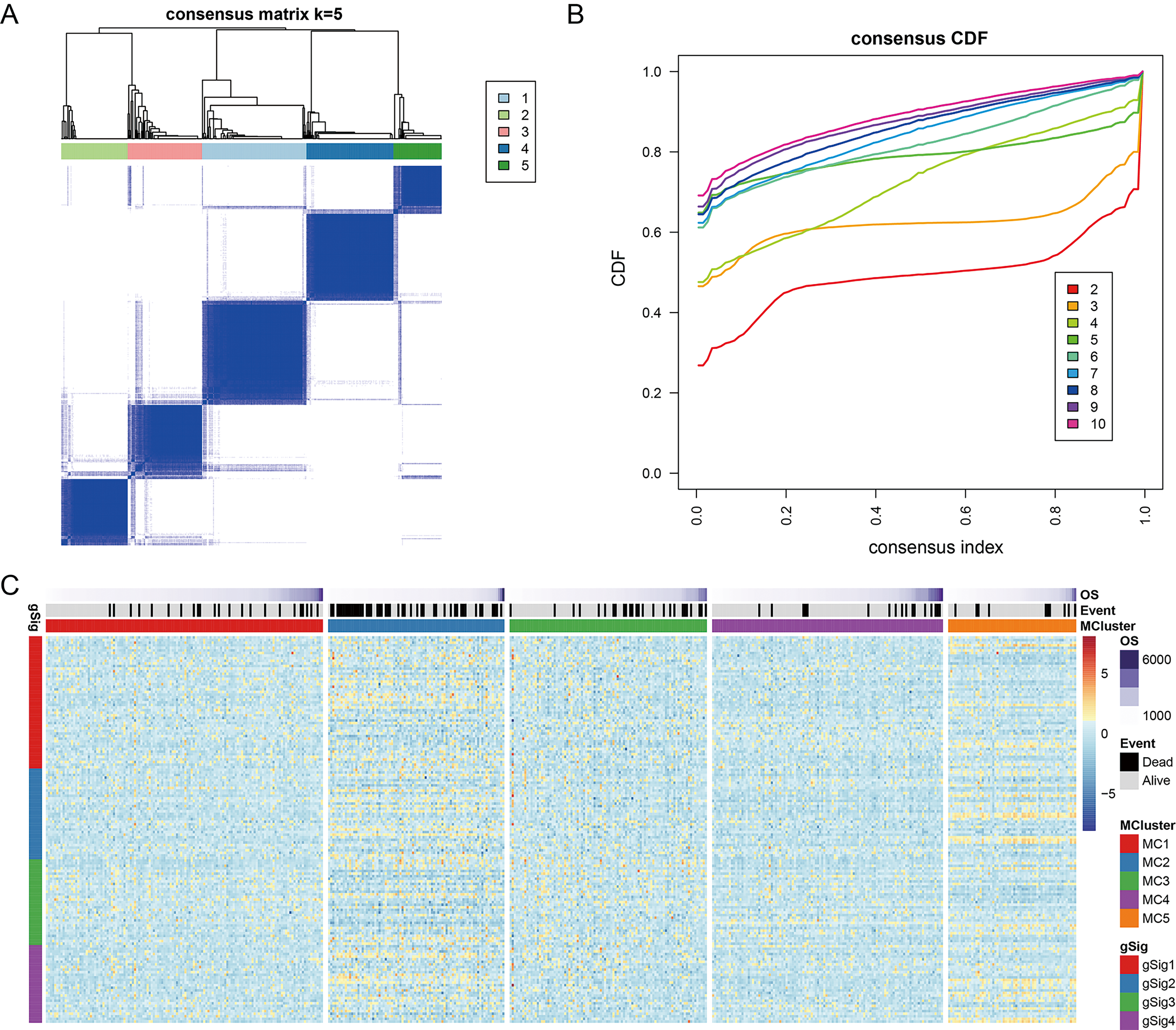

Supplement: Supplemental Material [file KCBT_A_2255369_SM1060.zip › Supplementary material/S8_Fig.png]

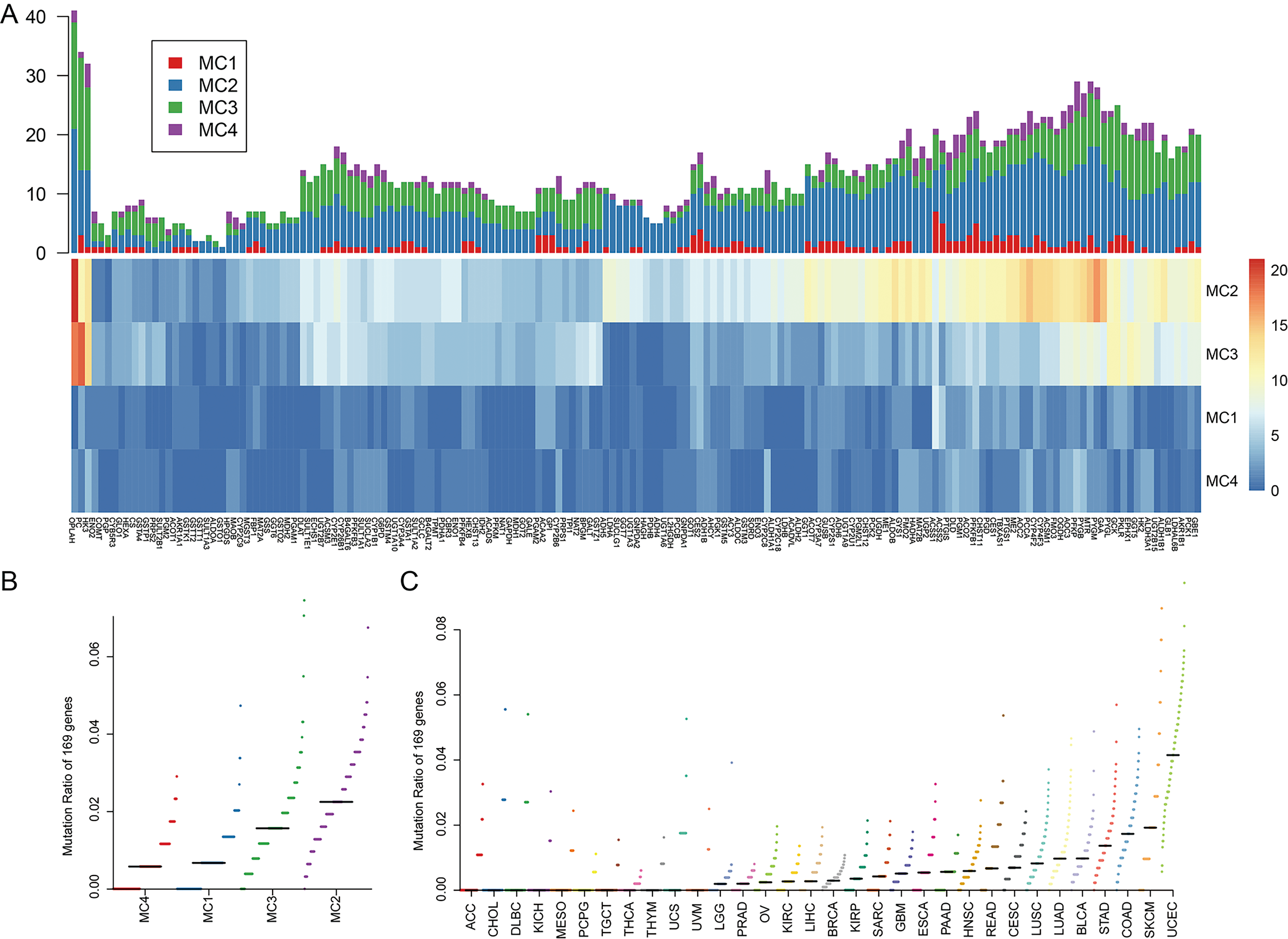

Supplement: Supplemental Material [file KCBT_A_2255369_SM1060.zip › Supplementary material/S9_Fig.png]
